# Evolution of codon usage in Zika virus genomes is host and vector specific

**DOI:** 10.1038/emi.2016.106

**Published:** 2016-10-12

**Authors:** Azeem Mehmood Butt, Izza Nasrullah, Raheel Qamar, Yigang Tong

**Affiliations:** 1 Genomics Laboratory, Department of Biosciences, COMSATS Institute of Information Technology, Islamabad 45550, Pakistan; 2 State Key Laboratory of Pathogen and Biosecurity, Beijing Institute of Microbiology and Epidemiology, Beijing 100071, China

**Keywords:** codon usage, evolution, mutation pressure, natural selection, Zika virus

## Abstract

The codon usage patterns of viruses reflect the evolutionary changes that allow them to optimize their survival and adapt their fitness to the external environment and, most importantly, their hosts. Here we report the genotype-specific codon usage patterns of Zika virus (ZIKV) strains from the current and previous outbreaks. Several genotype-specific and common codon usage traits were noted in the ZIKV coding sequences, indicating their independent evolutionary origins from a common ancestor. The overall influence of natural selection was more profound than that of mutation pressure, acting on a specific set of viral genes in the Asian-genotype ZIKV strains from the recent outbreak. An interplay between codon adaptation and deoptimization may have allowed the virus to adapt to multiple host and vectors and is reported for the first time in ZIKV genomes. Combining our codon analysis with geographical data on *Aedes* populations in the Americas suggested that ZIKV has evolved host- and vector-specific codon usage patterns to maintain successful replication and transmission chains within multiple hosts and vectors.

## Introduction

Zika virus (ZIKV), a member of the genus *Flavivirus* in the family *Flaviviridae*, is an enveloped, single-stranded positive-sense RNA virus. The full-length ZIKV genome sequence contains 10 794 nucleotides encoding 3419 amino acids, two flanking untranslated regions (5′ and 3′ UTRs), and a single long open reading frame that encodes a polyprotein, which is cleaved into the capsid (C), precursor membrane (prM), envelope (E) and seven nonstructural (NS) proteins (5′-C–prM–E–NS1–NS2A–NS2B–NS3–NS4A–NS4B–NS5-3′).^1, 2^ Like the closely related dengue virus (DENV), ZIKA is also mosquito-borne and is spread by several *Aedes* species. Since its discovery in 1947, reports of ZIKV detection have been largely sporadic until the recent and ongoing outbreak of ZIKV infection in the Americas,^3^ with a few notable exceptions, including the ZIKV outbreaks in the Yap Islands (2007)^4^ and French Polynesia (2013–2014).^5^ The typical symptoms of ZIKV infection include fever, headache, joint pain, rashes and conjunctivitis. The close similarity between the clinical manifestations of ZIKV and those of DENV and Chikungunya virus (CHIKV) remains a potential obstacle to the efficient surveillance and management of ZIKV.

The genetic code is redundant, and most amino acids can be translated by more than one codon. This redundancy is a key factor modulating the efficiency and accuracy of protein production and maintaining the same amino-acid sequence of the protein. Alternative codons within the same group that encode the same amino acid are often called ‘synonymous' codons, although their corresponding tRNAs may differ in their relative abundances in cells and in the speed with which they are recognized by the ribosome. However, synonymous codons are not randomly selected within and between genomes, and this is referred to as ‘codon usage bias'.^6, 7^ This phenomenon has been observed in a wide range of organisms, from prokaryotes to eukaryotes and viruses. Studies of codon usage have identified several factors that can influence codon usage patterns, including mutation pressure, natural or translational selection, secondary protein structure, replication, selective transcription, hydrophobicity and hydrophilicity of the protein, and the external environment.^8, 9, 10, 11, 12, 13^ When the size of the viral genome and other viral features, such as its dependence on the host machinery for key processes (including replication, protein synthesis and transmission) are compared with those of prokaryotic and eukaryotic genomes, the interplay between the codon usage of the virus and that of its host is expected to affect the overall viral survival, fitness, evasion of the host immune system and evolution.^11, 14^ Therefore, knowledge of the codon usage of viruses can provide information about their molecular evolution, extend our understanding of the regulation of viral gene expression, and improve vaccine design, for which the efficient expression of viral proteins may be required to generate immunity.

In a recent *in silico* analysis, Freire *et al.* reported that the codon adaptation of the ZIKV *NS1* gene is more biased toward *H. sapiens* than toward *Ae. aegypti*, and suggested that this could improve the replication of the virus within human cells.^15^ This is an interesting aspect of ZIKV evolution, but in that study, the codon adaptation indices were only calculated for *H. sapiens* and *Ae. aegypti* and lacked some of the recently sequenced ZIKV genomes from outbreak regions, which were not available at the time of the study. ZIKV is carried and transmitted by many types of *Aedes* mosquitoes other than *Ae. aegypti*, most notably *Ae. albopictus*,^4^ and understanding the evolutionary adaptation of the virus to its hosts and vectors is essential for developing effective surveillance, diagnostic, and preventive strategies. Based on recently available sequence data from the current outbreak and the broad range of the ZIKV vectors, we investigated the evolutionary adaptation of ZIKV to its transmission vectors and host to identify the potential cause of the ongoing massive ZIKV outbreak.

## Materials and methods

### Data analyzed

The complete genome sequences and coding sequence annotations of 31 ZIKV strains available at the time of study were obtained from the National Center for Biotechnology (NCBI) GenBank database^16^ and the Virus Pathogen Resource database,^17^ accessed on 10 February 2016. The demographics of the selected strains are given in Supplementary Table S1.

### Recombination and phylogenetic analyses

Potential recombination events in the ZIKV genomes were identified with the Recombination Detection Program (version 4.70) software suite,^18^ which incorporates multiple phylogenetic-substitution- and distance-based methods. The *P*-value cutoff was set to 0.05 in all analyses, and the Bonferroni correction was applied. The default settings were used for all analyses. Phylogenetic analysis and tree construction was performed using MEGA (version 7.0),^19^ applying the maximum likelihood model (ML) with bootstrap replicates. The best-fit nucleotide substitution model was selected by the Akaike information criterion implemented in MEGA7.

### Nucleotide composition analysis

The following compositional properties were calculated for the coding sequences of the ZIKV genomes: (i) overall frequency of occurrence of nucleotides (A%, C%, T/U% and G%); (ii) frequency of each nucleotide at the third site of synonymous codons (A_3_%, C_3_%, U_3_% and G_3_%); (iii) frequencies of occurrence of nucleotides G+C at the first (GC_1_), second (GC_2_) and third synonymous codon positions (GC_3_); (iv) mean frequencies of nucleotides G+C at the first and second positions (GC_1,2_); and (v) overall GC and AU contents. The codons AUG and UGG are the only codons for Met and Trp, respectively, and the termination codons UAA, UAG and UGA do not encode any amino acids. Therefore, these five codons are not expected to show any usage bias and were excluded from the analysis.

### Relative synonymous codon usage analysis

The relative synonymous codon usage (RSCU) values for all the coding sequences of the ZIKV genomes were calculated to determine the characteristics of synonymous codon usage without the confounding influences of the amino-acid compositions or the coding sequence sizes of different gene samples. The RSCU index was calculated as follows:


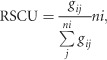


where *g*_*ij*_ is the observed number of the *i*th codon for the *j*th amino acid, which has *n*_*i*_ kinds of synonymous codons.^20^ The RSCU values represent the ratio between the observed usage frequency of one codon in a gene sample and the expected usage frequency in the synonymous codon family, given that all codons for the particular amino acid are used equally. Synonymous codons with RSCU values >1.0 show positive codon usage bias and were defined as ‘abundant' codons, whereas those with RSCU values <1.0 show negative codon usage bias and were defined as ‘less-abundant' codons. When the RSCU value is 1.0, there is no codon usage bias for that amino acid and the codons are chosen equally or randomly.^21^ Synonymous codons with RSCU values >1.6 and <0.6 were treated as ‘overrepresented' and ‘underrepresented' codons, respectively.^22^

### Effective number of codons analysis

An effective number of codons (ENC) analysis was used to quantify the absolute codon usage bias by evaluating the degree of codon usage bias displayed by the ZIKV coding sequences, regardless of the gene lengths and the numbers of amino acids. ENC values range from 20, indicating extreme codon usage bias and the use of only one of the possible synonymous codons for the corresponding amino acid, to 61, indicating no bias but the use of all possible synonymous codons equally for the corresponding amino acid. Thus, the larger the extent of codon preference in a gene, the smaller the ENC value. It is also generally accepted that genes have significant codon bias when the ENC value is ≤35.^23, 24^ ENC was calculated with the formula:


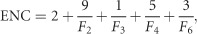


where 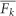 (*k*=2, 3, 4, 6) is the mean *F*_*k*_ values for *k*-fold degenerate amino acids, which is estimated with the formulae:


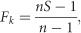


where *n* is the total number of occurrences of the codon for that amino acid and


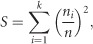


where *n*_*i*_ is the total number of occurrences of the *i*th codon for that amino acid. Genes for which the codon choice is only constrained by mutation bias are considered to lie on or just below the curve of the expected ENC values. Therefore, to clarify the relationship between the GC_3_ and ENC values, the expected ENC values for different GC_3_ were calculated as follows:


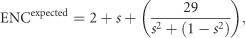


where *s* represents the given GC_3_%.

### Correspondence analysis

Correspondence analysis (CA) is a multivariate statistical method that was used to analyze the major trends in the codon usage patterns among the ZIKV coding sequences. To minimize the effect of the amino-acid composition on codon usage, each coding sequence was represented as a 59-dimensional vector, and each dimension corresponded to the RSCU value for each sense codon. These only included synonymous codons for a particular amino acid, and excluded codons AUG, UGG and the three stop codons.

### Parity rule 2 analysis

The Parity rule 2 (PR2) plot analysis was performed to investigate the effects of mutation and natural selection on the codon usage of individual genes. PR2 is a plot in which the AU-bias [A_3_/(A_3_+U_3_)] at the third codon position of the four-codon amino acids of entire genes is the ordinate and the GC-bias [G_3_/(G_3_+C_3_)] is the abscissa. The center of the plot, where both coordinates are 0.5, is where A=U and G=C (PR2), with no bias between the influence of the mutation and selection rates (substitution rates).^25, 26^

### Neutrality plot analysis

The neutrality plot or neutral evolution analysis was used to determine and compare the extent of the influences of mutation pressure and natural selection on the codon usage patterns of the ZIKV coding sequences by plotting the *P*_12_ (GC_1,2_) values of the synonymous codons against the *P*_3_ (GC_3_) values. In this plot, the regression coefficient against *P*_3_ is regarded as the mutation-selection equilibrium coefficient and the evolutionary rates of the mutation pressure and natural selection pressure are expressed as the slopes of the regression lines. If all of the points lie along the diagonal distribution, no significant difference exists at the three codon positions, and there is no or weak external selection pressure. Alternatively, if the regression curve tends to be sloped or parallel to the horizontal axis, the variation correlation between GC_1,2_ and GC_3_ is very low. Therefore, the regression curve effectively measures the degree of neutrality when selecting the effect that dominates evolution.^27^

### Codon adaptation index

Codon adaptation index (CAI) is a quantitative measure that predicts the expression level of a gene based on its coding sequence. CAI values range from 0 to 1. The most frequent codons show the highest relative adaptation to the host, and sequences with higher CAIs are considered to be preferred over those with lower CAIs.^28^ The CAI analysis of the ZIKV coding sequences was performed with the CAIcal server,^29^ which implements an improved CAI calculation method. The synonymous codon usage patterns of *H. sapiens*, *Ae. aegypti* and *Ae. albopictus* were used as references. Nonsynonymous codons and termination codons were excluded from the calculation. The reference data sets were obtained from the Codon Usage Database (CUD).^30^

### Relative codon deoptimization index

The relative codon deoptimization index (RCDI) values for the ZIKV coding sequences were calculated to determine the codon deoptimization trends by comparing the similarity in codon usage of a given coding sequence with that of a reference genome using the RCDI/eRCDI server. An RCDI value of 1 indicates that the virus follows the host codon usage pattern and displays a host-adapted codon usage pattern. Conversely, RCDI values higher than 1 indicate the deoptimization of the codon usage patterns of the virus from that of its host(s).^31, 32^ The synonymous codon usage patterns for *H. sapiens*, *Ae. aegypti* and *Ae. albopictus* were used as references and were obtained from the CUD. Nonsynonymous codons and termination codons were excluded from the calculation.

### Similarity index

The influence of the overall codon usage pattern of the host on the formation of the overall codon usage of the virus, defined as the similarity index (SiD), was calculated as follows:


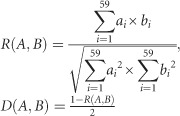


where *R*(*A*,*B*) is defined as the cosine value of the angle included between the *A* and *B* spatial vectors, and represents the degree of similarity between the ZIKV and host overall codon usage patterns. *a*_*i*_ is defined as the RSCU value for a specific codon among the 59 synonymous codons of the ZIKV coding sequence. *b*_*i*_ is the RSCU value for the same codon in the host. *D*(*A*,*B*) represents the potential effect of the overall codon usage of the host on that of ZIKV, and its value ranges from 0 to 1.0.^33^

### Correlation analysis

A correlation analysis was performed to identify the relationships between the nucleotide composition, CA and the codon usage patterns of ZIKV using Spearman's rank correlation analysis. All statistical analyses were performed with SPSS 23 (SPSS Inc., Chicago, IL, USA).

## Results

### Recombination and phylogenetic analyses of ZIKV genomes

To avoid the effects of recombination artifacts on the overall codon usage patterns and the topology of the phylogenetic tree, the ZIKV genomes were first scanned for the presence of potential recombinants. Four of 31 ZIKV genomes were found to be recombinants (Supplementary Table S1) and were excluded from further analysis. The remaining 27 ZIKV genomes were subjected to a phylogenetic analysis to infer the genotype-specific codon usage patterns, particularly those of the Asian genotype (Supplementary Table S1). A ML tree, verified with 1000 bootstrap replicates, was constructed with the TN93+G model. The ZIKV isolates clustered into three genotypic groups: Asian (*n*=21), East African (EA; *n*=4) and West African (WA; *n*=2; Supplementary Figure S1).

### G and A nucleotides are more frequent than C and U in ZIKV coding sequences

To determine the potential influence of compositional constraints on codon usage, the nucleotide compositions of the ZIKV coding sequences were determined. The mean compositions (%) of nucleotides G (29.19±0.16%) and A (26.76±0.17%) were highest, followed by U (22.11±0.20%) and C (21.94±0.20%). The nucleotides at the third positions of synonymous codons (A_3_, U_3_, G_3_ and C_3_) showed similar compositional trends as the mean values: G_3_ (30.32±0.53%) and A_3_ (25.13±0.52%) were also higher than C_3_ (25.09±0.47%) and U_3_ (19.46±0.51%). The mean GC and AU compositions were 51.13±0.35% and 48.87±0.35%, respectively, whereas the mean GC_3_ and AU_3_ compositions were 55.41±0.97% and 44.59±0.97%, respectively. According to the nucleotide occurrence frequencies at the third positions of codons, the ZIKV coding sequences were GC-rich. However, at an individual nucleotide level, G and A predominated over C and U (Supplementary Table S2). Significant differences (*P*<0.05) were also observed in the mean GC, AU, GC_3_ and AU_3_ values for the ZIKV isolates, according to the genotypic classification, indicating that the compositional patterns of the ZIKV coding sequences are more complex than the commonly observed GC- and/or AU-rich compositions of most viruses.

### Codon usage bias among ZIKV coding sequences varies and is genotype-specific

**Figure 1:**
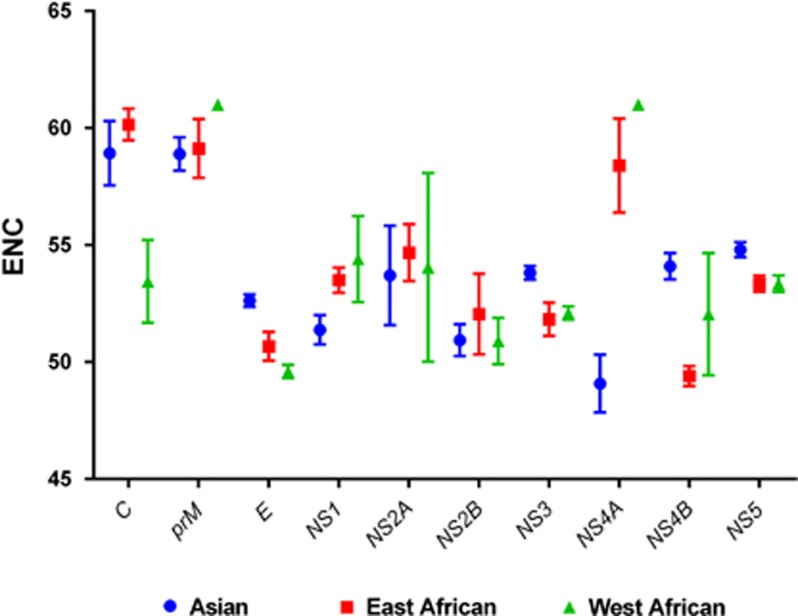
Genotype-specific comparative analysis of ENC values of ZIKV coding sequences.

To estimate the magnitude of the codon usage bias within the ZIKV coding sequences, the ENC values were computed. A mean value of 53.93±0.39 was obtained for the complete coding sequences of all strains, irrespective of genotype. Individually, the highest ENC value was for the *prM* (59.08±0.94) and the lowest for the *NS2B* (51.1±0.95) coding sequences. A mean ENC value of 53.83±0.37 was obtained when only ZIKV strains of the Asian genotype were analyzed. The highest ENC value was obtained for the *C* (58.92±1.37) and the lowest for the *NS4A* (49.08±1.23) coding sequences in the Asian genotype (Figure 1). Significant differences (*P*<0.05) were observed in the mean ENC values for the *C*, *E*, *NS1*, *NS3*, *NS4A*, *NS4B* and *NS5* coding sequences when the genotypic classification of the ZIKV isolates was considered. Overall, the mean ENC values suggested a relatively conserved and genotype-specific evolution of codon usage bias within the ZIKV coding sequences.

### ZIKV genes have evolved genotype- and host-specific RSCU patterns

An RSCU analysis was performed to determine the patterns of and preferences for synonymous codons in the ZIKV coding sequences. Among the 18 most abundantly used codons, 11 were G/C-ended (seven C-ended; four G-ended) and the remaining seven were A/U-ended (six A-ended; one U-ended) when the ZIKV coding sequences were not differentiated according to their genotypic group. This shows that C- and A-ended codons are preferred in the ZIKV coding sequences. An analysis of over- and underrepresented codons, irrespective of the ZIKV genotype, showed that 5 of the 18 preferred codons (CUG [Leu], GUG [Val], CCA [Pro], AGA [Arg] and GGA [Gly]) had RSCU values >1.6, whereas the remaining preferred codons had RSCU values >0.6 and <1.6. An estimate of the overall RSCU can potentially hide genotype-specific patterns, so we next calculated the RSCU values of the ZIKV coding sequences according to the genotypic groups. We noted that the preferred codons varied among the genotypic groups. The ratios of common/uncommon preferred codons between the Asian:EA, Asian:WA and EA:WA genotypes were 16:2, 15:3 and 13:5, respectively. Patterns of genotype-specific codon overrepresentation were also observed: among the ZIKV isolates, 5 of the 18 preferred codons were overrepresented in the Asian genotype, 7 of the 18 were overrepresented in the EA genotype and 5 of the 18 were overrepresented in the WA genotype. None of the preferred codons were underrepresented (RSCU<0.6), regardless of whether the ZIKV strains were classified into their respective genotypes. The genotype-specific RSCU patterns highlight the independent evolutionary dynamics of the ZIKV isolates. Therefore, to determine the potential influences of the host and vectors on the codon usage patterns of the ZIKV isolates, the RSCU patterns of the ZIKV coding sequences were correlated with those of *H. sapiens*, *Ae. aegypti* and *Ae. albopictus*. Interestingly, a mixture of coincidence and antagonism was observed in the codon usage patterns as ZIKV showed no complete coincidence or complete antagonism to any of the patterns of its host and vectors. Among the 18 preferred codons, the ratio of coincident/antagonist preferred codons was 11/7 between ZIKV and *H. sapiens*; 10/8 between ZIKV and *Ae. aegypti*; and 9/9 between ZIKV and *Ae. albopictus* (Table 1).

### Trends in codon usage variations

**Figure 2:**
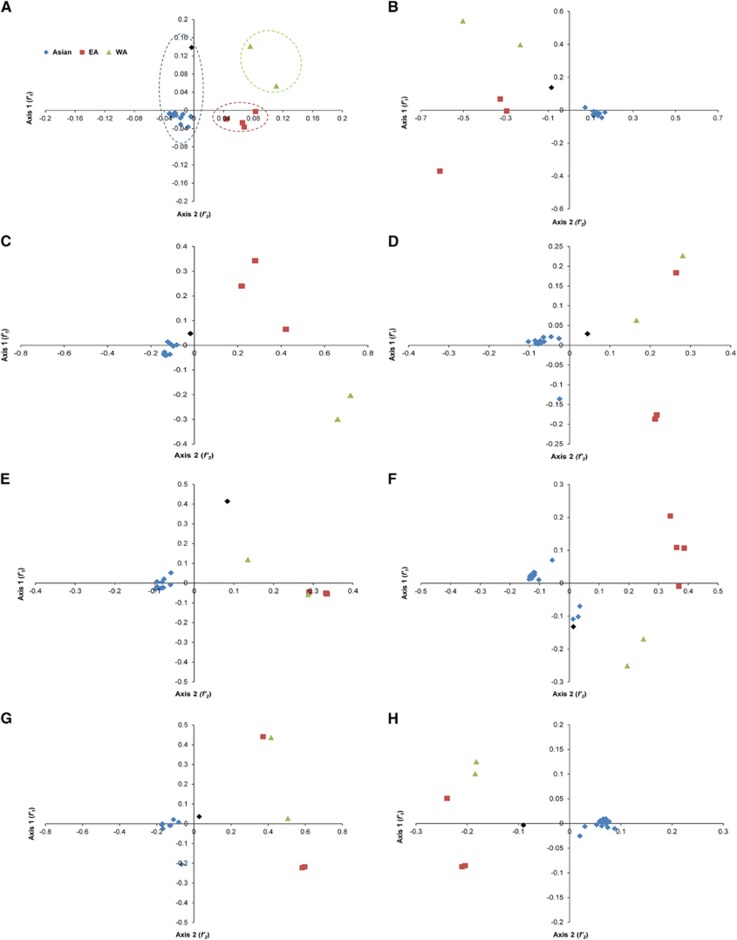

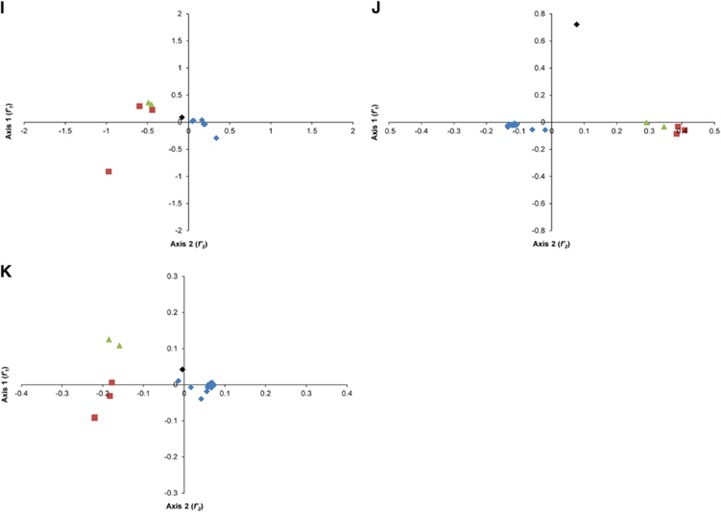
Correspondence analysis (CA). Genotype-specific CA plots were constructed for whole genome and individual ZIKV coding sequences. (**A**) Whole genome. (**B**) *C*. (**C**) *prM*. (**D**) *E*. (**E**) *NS1*. (**F**) *NS2A*. (**G**) *NS2B*. (**H**) *NS3*. (**I**) *NS4A*. (**J**) *NS4B*. (**K**) *NS5*. The description of color coding is same as that of **A**. The ZIKV strain isolated from *Ae. aegypti* is represented as a black color diamond.

To determine the variations in the synonymous codon usage among the coding sequences of different ZIKV strains, CA plots of the combined full-length coding sequences and of individual coding sequences were constructed. On average, the first (*f'*_1_) and second principal (*f'*_2_) axes accounted for 54.1% and 17.2% of the total variation, respectively, indicating that *f'*_1_ accounts for the major proportion of codon usage variations. The ZIKV strains grouped into three well-defined clusters on the axes plots, where cluster I contained 21 strains, cluster II contained two strains, and cluster III contained four strains. When we investigated this variation according to the distribution of genotypes, the strains clustered consistently with the phylogenetic analysis reported in the present study. All the Asian genotype strains grouped into cluster I; cluster II consisted of WA strains; and cluster III consisted of EA strains. The ZIKV isolates from the recent outbreak and that from French Polynesia grouped into cluster I. At the individual gene level, the genotype-specific clustering was observed to be stronger among the Asian strains than among the WA and EA genotypes, with some exceptions. For instance, the coding sequences of ZIKV (Genbank: HQ234499) isolated from *Ae. aegypti* did not cluster closely with the other Asian strains, highlighting the different trends in codon usage in ZIKV from different hosts (Figure 2A). In contrast, in the analyses of some genes, such as those encoding the C, NS2, NS5 and prM proteins, the WA and EA strains clustered independently, whereas in the analyses of the genes encoding E, NS1, NS2B and NS4A, overlapping trends in codon usage were observed. These data indicate a common ancestry, but the occurrence of independent divergence events at the level of individual genes (Figures 2B–2K).

### Mutation pressure and natural selection have both influenced codon usage patterns in ZIKV

**Figure 3:**
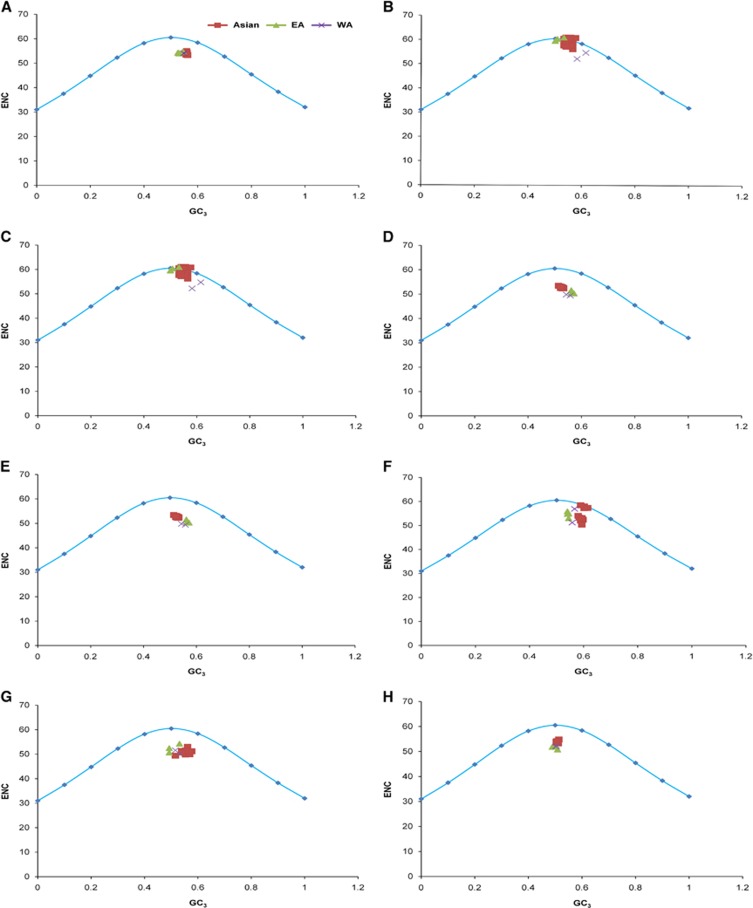

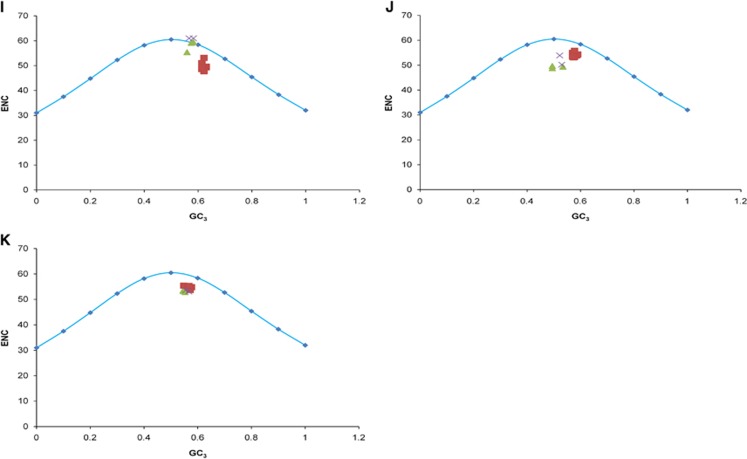
ENC–GC_3_ plots. The curve indicates the expected codon usage if GC compositional constraints alone account for the codon usage bias. (**A**) Whole genome. (**B**) *C*. (**C**) *prM*. (**D**) *E*. (**E**) *NS1*. (**F**) *NS2A*. (**G**) *NS2B*. (**H**) *NS3*. (**I**) *NS4A*. (**J**) *NS4B*. (**K**) *NS5*. The description of color coding is same as that of **A**.

To determine whether the codon usage patterns of the ZIKV coding sequences have been shaped solely by mutation pressure, natural selection or both, we performed a correlation analysis of the nucleotide compositions, codon compositions and principal axes, and then constructed PR2 bias and ENC–GC_3_ plots. As given in Supplementary Table S3, a mixture of significant and nonsignificant correlations was observed between several indices and the principal axes, indicating the influence of both mutation pressure and natural selection. The majority of compositional constraints correlated significantly with *f'*_1_. To determine whether the biased codon choices were restricted to highly biased protein-coding genes, the relationships between the A–U contents and the G–C contents in the fourfold degenerate codon families (Ala, Arg, Gly, Leu, Pro, Ser, Thr and Val) were analyzed with a PR2 plot. We found that A and G were used more frequently than U and C in the fourfold degenerate codon families in the ZIKV coding sequences (Supplementary Figure S2). This unequal use of nucleotides suggests the overlapping influences of natural selection and mutation pressure on the codon preferences in the ZIKV coding sequences. To further clarify the effects of mutation pressure and natural selection, ENC–GC_3_ plots were constructed for the whole polyprotein and individual coding sequences. In the plot of the whole polyprotein sequences, the ZIKV strains from all genotypes clustered together below the expected ENC curve (Figure 3A). None of the strains fell on the expected curve, which would have indicated mutation pressure, whereas the below-curve clustering showed that the influence of natural selection dominated that of mutation pressure in the ZIKV strains. However, the influence of mutation pressure was not completely absent and the effects of mutation pressure and natural selection on individual coding sequences varied, even within a single strain, and also genotype specifically. For instance, the *C*, *prM* and *NS2A* coding sequences of some of the Asian genotype strains fell on the expected curve, showing the dominant influence of mutation pressure rather than natural selection. When investigated further, it was found that none of the mutation pressure influenced Asian genotype strains were from the recent outbreak and were those which were isolated during a period of 1966–2012 (Supplementary Table S1). In contrast, the remaining coding sequences in the same ZIKV strains did not follow the same pattern, but were strongly influenced by natural selection (Figures 3B–3K).

### Natural selection predominates in shaping the codon usage patterns in ZIKV

Once we established that both mutation pressure and natural selection have contributed to shaping the codon usage patterns of the ZIKV coding sequences, the magnitude of both forces was investigated, initially by constructing neutrality plots and then by calculating the CAI, RCDI and SiD.

In the neutrality plot analysis, a significant positive correlation was observed between the mean *P*_12_ (GC_1,2_) and *P*_3_ (GC_3_) values (*r*=0.50, *P*=0.007) of the ZIKV polyprotein sequences. However, the slope of the regression line was calculated to be 0.032, according to that the relative neutrality (mutation pressure) was 3.2%, and the relative constraint on GC_3_ (natural selection) was 96.8%, indicating the dominant influence of natural selection on the codon usage patterns of ZIKV. In the Asian-genotype ZIKV strains, the correlation between *P*_12_ and *P*_3_ was not significant (*P*>0.05), with a slope of −0.078, and the mutation pressure and natural selection were calculated to be 7.8% and 92.2%, respectively, again demonstrating the dominant influence of natural selection. Similarly, in the EA and WA strains, natural selection was also predominant over mutation pressure with slope values of 0.014 and 0.131, respectively (Supplementary Figure S3A). We then performed the neutrality plot analysis of individual coding sequences in the ZIKV strains of the Asian genotype. Although, there were significant correlations between *P*_12_ and *P*_3_ values of *C*, *E* and *NS1* coding sequences, regression slope values of each were closer to zero, indicating influence of natural selection, whereas the remaining genes had nonsignificant correlations between *P*_12_ and *P*_3_ values (Supplementary Figures S3B–S3K). Overall influence of natural selection remained predominant on coding sequences of ZIKV strains based on near-zero or negative regression slopes and nonsignificant correlations between *P*_12_ and *P*_3_ values.

### ZIKV displays host-specific codon adaption patterns

**Figure 4:**
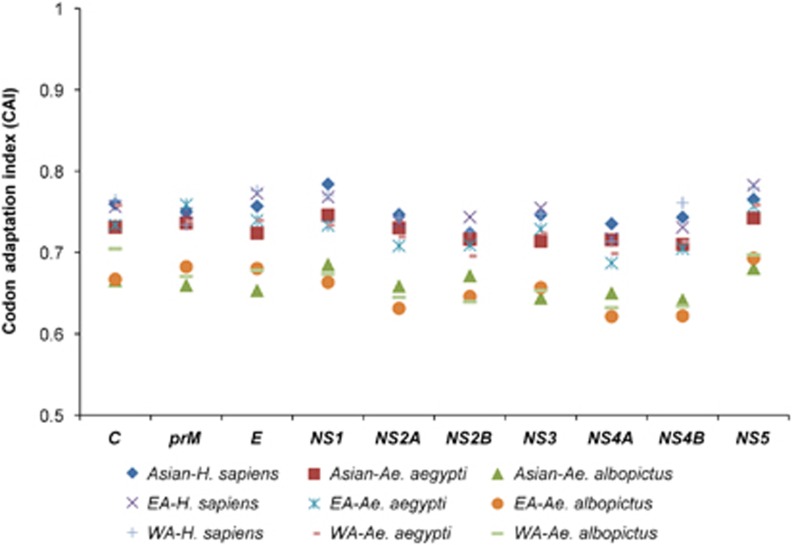
CAI analysis of the ZIKV coding sequences in relation to its host and vectors. East African, EA; west African, WA.

A CAI analysis was performed to determine the correlation between the codon usage bias and the expression levels of the ZIKV coding sequences, which reflects the adaptation of the viral genes to the host cellular machinery. Mean CAI values of 0.75±0.001, 0.72±0.001 and 0.66±0.002 were obtained for the ZIKV polyproteins in relation to *H. sapiens*, *Ae. aegypti* and *Ae. albopictus*, respectively. The CAI values were then calculated for individual coding sequences in relation to *H. sapiens*, *Ae. aegypti* and *Ae. albopictus*, according to the genotypic classification of the ZIKV strains. In the ZIKV isolates of the Asian genotype, the highest mean CAI was noted for *NS1* in relation to *H. sapiens* (0.78±0.003), *Ae. aegypti* (0.75±0.002) and *Ae. albopictus* (0.68±0.002). In the EA strains, the highest mean CAI was noted for *NS5* in relation to *H. sapiens* (0.78±0.003) and *Ae. albopictus* (0.68±0.002), whereas the *NS5* (0.76±0.003) and *prM* (0.76±0.001) coding sequences both had the highest CAI values in relation to *Ae. aegypti*. In the WA strains, the highest mean CAI was noted for *NS5* in relation to *H. sapiens* (0.78±0.002) and *Ae. aegypti* (0.76±0.003), whereas *C* had the highest mean CAI in relation to *Ae. albopictus* (0.70±0.05) (Figure 4 and Supplementary Table S4).

### ZIKV displays the highest codon usage deoptimization for *Ae. albopictus*

**Figure 5:**
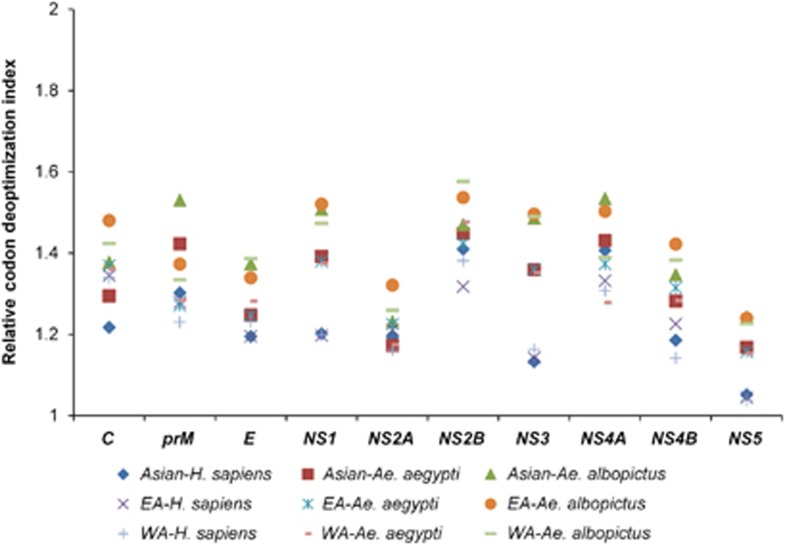
RCDI analysis of the ZIKV coding sequences in relation to its host and vectors. East African, EA; west African, WA.

The RCDI values were calculated to determine the codon usage deoptimization in the ZIKV coding sequences in relation to *H. sapiens*, *Ae. aegypti* and *Ae. albopictus*. Mean RCDI values of 1.23±0.008, 1.32±0.011 and 1.41±0.012 were obtained in relation to *H. sapiens*, *Ae. aegypti* and *Ae. albopictus*, respectively, for the complete polyprotein sequences, indicating the highest codon deoptimization in the ZIKV coding sequences in relation to *Ae. albopictus*. Furthermore, in the Asian-genotype isolates, the highest and lowest RCDI values in relation to *H. sapiens* were for *NS2B* (1.41±0.03) and *NS5* (1.05±0.006), respectively. The highest and lowest RCDI values in relation to *Ae. aegypti* were for *NS2B* (1.45±0.02) and *NS5* (1.17±0.006), respectively. In contrast to this, the highest and lowest RCDI values in relation to *Ae. albopictus* were of *NS4A* (1.53±0.03) and *NS2A* (1.23±0.013), respectively (Figure 5 and Supplementary Table S5).

### *Ae. albopictus* has induced stronger selection pressure on ZIKV

**Figure 6:**
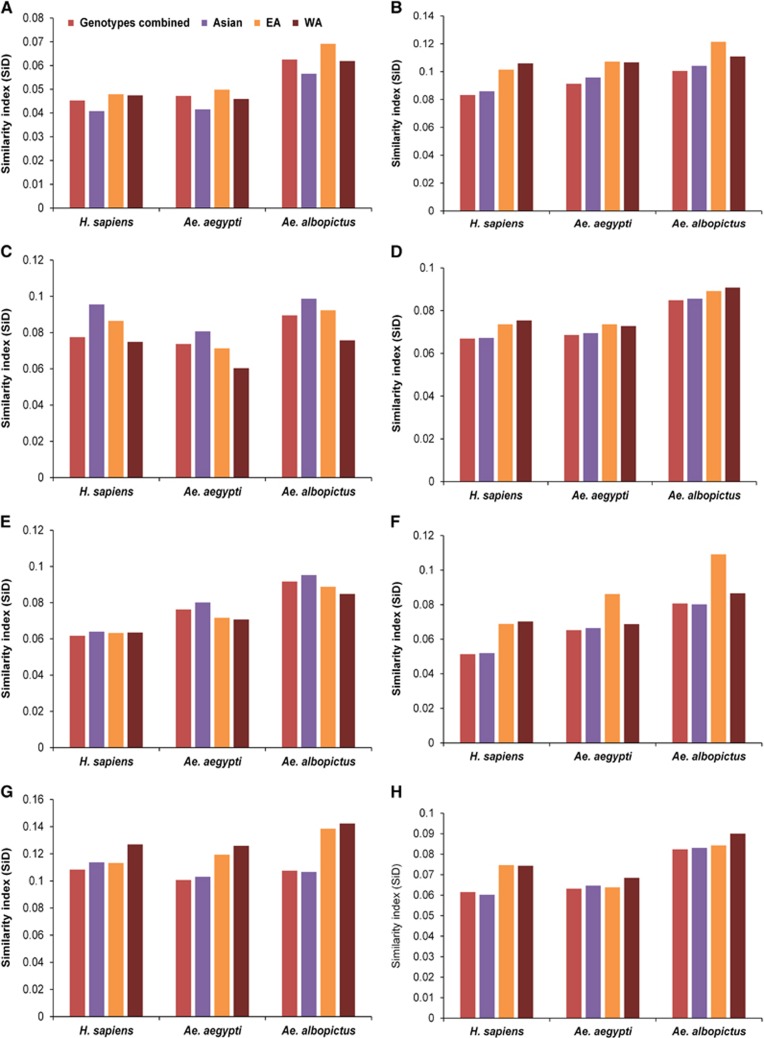

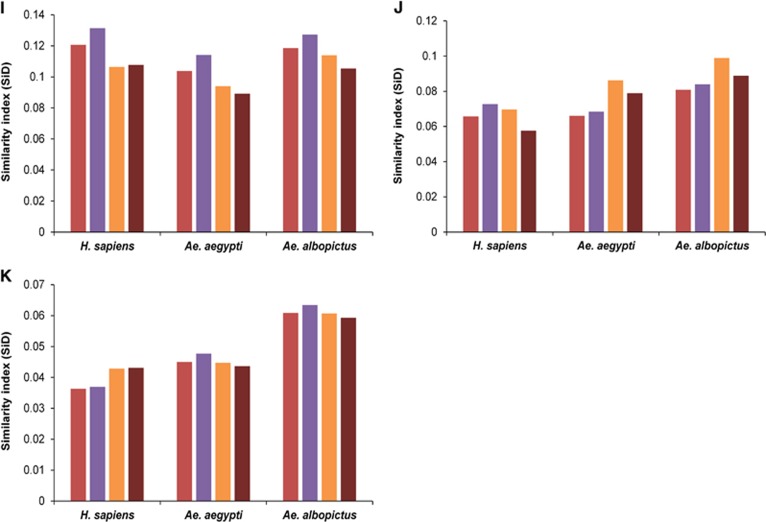
SiD analysis of ZIKV strains in relation to its host and vectors. (**A**) Whole genome. (**B**) *C*. (**C**) *prM*. (**D**) *E*. (**E**) *NS1*. (**F**) *NS2A*. (**G**) *NS2B*. (**H**) *NS3*. (**I**) *NS4A*. (**J**) *NS4B*. (**K**) *NS5*. The description of color coding is same as that of **A**.

To determine the potential influence of the codon usage patterns of *H. sapiens*, *Ae. aegypti* and *Ae. albopictus* on the evolution of the codon usage patterns of the ZIKV coding sequences, a SiD analysis was performed. We observed that *Ae. albopictus* exerted a greater effect on the ZIKV codon usage patterns than *Ae. aegypti* or *H. sapiens*, because the SiD was highest for *Ae. albopictus* versus the ZIKV group when the whole polyprotein sequences were considered, without the specification of the genotypes. When the polyprotein sequences were analyzed according to the genotypic classification, the trends in SiD values remained unchanged, which implies that all the genotypes of ZIKV have been most strongly influenced by *Ae. albopictus*. To determine whether the influences of the host and vectors were also similar for individual coding sequences, the SiD values were calculated for the individual coding sequences of the Asian-genotype ZIKV strains. Compared with the other coding sequences, *NS2B* and *NS4A* were more strongly influenced by *H. sapiens* than by *Ae. aegypti* or *Ae. albopictus*. Overall, the influence of *Ae. albopictus* was greater than that of *Ae. aegypti* or *H. sapiens* on the ZIKV coding sequences (Figures 6A–6K).

## Discussion

In the present study, we analyzed the codon usage patterns of ZIKV genomes from earlier and current epidemic periods to understand their evolutionary patterns. A genotype-specific codon usage analysis workflow similar to those reported previously by us for CHIKV^34^ was followed, with a particular focus on recently reported epidemic-associated ZIKV strains. Recombination events are known to influence codon usage patterns and the topologies of phylogenetic analyses and may lead to incorrect interpretations.^35, 36, 37, 38^ Therefore, recombinant ZIKV strains were first excluded from the present analysis, resulting in a final dataset of 27 full-length ZIKV genome sequences. According to a phylogenetic analysis, the ZIKV genomes clustered into three groups representing the Asian, EA, and WA genotypes. All the ZIKV strains isolated during the present ongoing epidemic clustered within the Asian genotype group. This is consistent with a previously reported phylogenetic grouping of ZIKV strains.^39^ The results of the CA analysis were also consistent with the phylogenetic analysis, because the ZIKV strains formed three clusters based on their genotypic classifications. However, at the level of individual genes, the clustering overlapped between strains of different genotypes, indicating that the ZIKV strains underwent evolutionary divergence from a common ancestor.

Codon usage bias, which results from the balance between mutational and translational/natural selection, is a widespread phenomenon across the genomes of several organisms and is reported to be profoundly influenced by genomic evolution. The extent of codon usage bias tends to be specific in individual sets of genes within a genome and is shaped by multiple factors. Here we quantified the influence of both mutation and natural selection on the coding sequences of the ZIKV strains in a stepwise manner, starting from a general assumption and leading to the selection of definite evolutionary factors. To start with, an ENC and nucleotide composition analysis was performed. In ZIKV, the ENC values were first calculated to estimate the overall codon usage bias in the whole viral genomes and in individual genes according to the genotypic classification. The overall codon usage bias was low among the genes of the ZIKV strains (ENC, 53.93). Low codon usage bias has also been observed among several other RNA viruses, such as Ebola virus (ENC, 57.23),^40^ CHIKV (ENC, 55.56)^34^ and hepatitis C virus (ENC, 52.62).^41^ It has been suggested that the low codon bias of RNA viruses is an advantage for their efficient replication in host cells by reducing the competition between the virus and the host for the synthesis machinery, which potentially have distinct codon preferences. Because the host and vectors of ZIKV have different codon usage preferences, the evolution of a low codon usage bias may have favored the maintenance of successful replication and transmission cycles in ZIKV. Although ENC values can be used to estimate the overall codon usage bias, these values alone are unable to show whether the underlying cause of codon bias is mutation pressure and/or natural selection. Codon usage bias, or a preference for one type of codon over another, can be greatly influenced by the overall nucleotide composition, and an ENC plot of genes whose codon choice is only constrained by a G_3_+C_3_ mutational bias will lie on or just below the continuous curve of the predicted ENC values.^42^ When present, this effect of nucleotide constraints reflects the dominant influence of mutation pressure. In ZIKV, we observed GC content to be comparatively higher than AU content in overall genomic composition. To determine how this genomic composition may have influenced the viral codon usage patterns, we derived our initial assumption from ENC–GC_3_ and PR2 analyses. These showed that both mutation pressure natural selection have influenced the codon usage patterns of the ZIKV coding sequences.

For several organisms, including viruses, a GC- or AU-rich composition tends to correlate with their RSCU patterns. For instance, a GC- or AU-rich genome tends to contain codons preferentially ending with either G and C or A and U, respectively. Such trends, when present, support the influence of mutation pressure. Surprisingly, in ZIKV, despite higher percentage of GC versus AU, the preferred codons end in C or A, rather than in GC or AU. Moreover, the selection of optimal codons also varies between the different genotypes. Because the selection of optimal codons in parasitic organisms, such as viruses, depends largely upon their hosts, we next compared the RSCU patterns of ZIKV with those of its host and vectors. Unlike several other viruses that have evolved either completely identical or opposite patterns of codon usage to their hosts,^32, 43^ ZIKV has evolved a mixture of coincident and antagonistic codon usage patterns relative to its host and vectors. A similar pattern of mixed codon preferences has also been detected in CHIKV, which exploits the same host and vectors as ZIKV.^34^ These results indicate that the selection pressure exerted by its host and vectors has greatly influenced the codon usage patterns and the possible fitness of ZIKV, adapting it to a dynamic range of hosts and vectors. It has also been suggested that a coincident codon usage between viruses and their hosts allows the corresponding amino acids to be translated efficiently, whereas an antagonistic codon usage may allow viral proteins to be folded properly, although the translation efficiency of the corresponding amino acids might be reduced.^41^

When the findings of the ENC, PR2 and RSCU analyses are combined, it is clear that both mutation pressure and natural selection have played roles in shaping the codon usage patterns of ZIKV. The significant correlation between the *P*_12_ and *P*_3_ values and the slope of the regression line close to 1 indicate that mutation pressure has been the main force, whereas the nonsignificant correlation between the *P*_12_ and *P*_3_ values and a slope of the regression line close to 0 are indicative of prevailing natural selection. To identify one force as the most influential factor, we calculated their influences by constructing neutrality plots between the *P*_12_ and *P*_3_ values of individual coding sequences. The nonsignificant correlations, nearly zero or less than zero slope values and higher values of the relative constraints compared with the relative neutrality were noted for the ZIKV coding sequences, confirming the predominant influence of natural selection on the codon usage patterns of the ZIKV strains of the Asian genotype. To further confirm the influence of natural selection, a CAI analysis was performed. CAI is frequently used as a measure of gene expression and to assess the adaptation of viral genes to their hosts, which reflects the influence of natural selection. It has been postulated that highly expressed genes display a stronger bias for particular codons than genes that are less frequently expressed. If the CAI value is high, then the codon usage bias is extremely high and the influence of natural selection is prevalent, and *vice versa*.^44^ On the basis of the CAI values for the ZIKV coding sequences, variable levels of adaptation to the ZIKV host and vectors were observed. On average, the greatest adaptation of ZIKV was to *H. sapiens*, closely followed by *Ae. aegypti* and then *Ae. albopictus*. The CAI values for the ZIKV coding sequences tended to be lower for *Ae. aegypti* and *Ae. albopictus*, which could be attributable to the potentially lower efficiency of protein synthesis in both vectors. On the contrary, the CAI values of all the ZIKV coding sequences in the Asian genotype were high for *H. sapiens*, suggesting that the replication of viral proteins could be more efficient within the host cells than within the vector cells.

Summarizing the observations made with the multiple codon usage indices used in present study, the ZIKV coding sequences are significantly influenced by host- and vector-induced natural selection, whereas mutation pressure has only a minor role. Interestingly, both forces have influenced different coding sequences differently, even within a single strain at the same time. For instance, when a neutrality plot analysis was conducted of the complete polyprotein sequences, irrespective of the genotypic classification, natural selection was found to be the dominant factor overall, rather than mutation pressure, in shaping the codon usage patterns. However, in the ZIKV coding sequences of the Asian genotype, mutation pressure most strongly influenced *NS3* rather than the other genes, which were strong influenced by natural selection. Similar observations were also made for the ZIKV strains of the EA and WA genotypes. This indicates that the evolution of the codon usage patterns of individual coding sequences is potentially associated with the function of each coding sequence in viral pathogenesis. While this manuscript was under-review, van Hemert *et al.* reported codon usage analysis of ZIKV genomes and suggested that biased nucleotide composition dictates the codon usage of ZIKV.^45^ As stated earlier, we also noted influence of mutation pressure that is indicative of biased nucleotide composition but unlike the findings of van Hemert *et al.*, we observed that natural selection is a predominant factor in ZIKV evolution. This difference could be due to certain limitations of Hemert *et al.* study such as; limited number of ZIKV genomes (*n*=9) were analyzed among which only four were from the recent ZIKV outbreak, ENC–GC_3_ plot was utilized as a major determining approach and other codon usage indices such as neutrality plots, PR2 bias, and CAI which better reflect interplay between mutation pressure and natural selection were not calculated. Most importantly, the analysis was conducted on complete ORF and not on individual coding sequences which might have masked the more accurate findings. In contrast, we analyzed a larger genome data set and at individual coding sequence level by incorporating multiple codon usage analysis approaches.

Recently, Freire *et al.* reported that the *NS1* gene in the Asian-genotype strains of the recent endemic was most strongly adapted to *H. sapiens*.^15^ In the CAI analysis of the present study, we also noted that the *NS1* gene was the gene most strongly adapted to *H. sapiens*, but unlike the findings of Freire *et al.*, high adaptation values for *NS1* were noted in all the ZIKV Asian-genotype strains from both the earlier and present endemic periods. We also observed similar CAI values for *NS1* in both *Ae. aegypti* and *H. sapiens*. Viruses such as ZIKV, DENV and CHIKV can replicate successfully in multiple hosts and vectors. Therefore, we propose that such multiple-host viruses must evolve a dynamic balance between codon adaptation and codon deoptimization to maintain efficient replication, survival, and pathogenic cycles in multiple hosts with various codon usage patterns. In such scenarios, we suspect that a single CAI observation is insufficient or potentially misleading when deciphering the evolutionary patterns of such multiple-host pathogens. Therefore, we incorporated two additional codon analysis indices, RCDI and SiD, in the present study to investigate the magnitude of ZIKV evolution under the influence of its host and vectors. According to the RCDI analysis, the ZIKV coding sequences had the highest RCDI values for *Ae. albopictus*, and the lowest RCDI values for *Ae. aegypti* and *H. sapiens*. As reported previously, a low RCDI might indicate strong adaptation to a host, which is consistent with the overall high CAI of the ZIKV coding sequences for *H. sapiens* and *Ae. aegypti*. In contrast, a high RCDI indicate that some viral genes are expressed in latency phases, or even that the virus presents a low replication rate for its successful establishment in a host with alternative codon usage patterns.^31^ Furthermore, as shown in the SiD analysis, *Ae. albopictus* is potentially the newly preferred vector of ZIKV because the selection pressure exerted by *Ae. albopictus* on the codon usage patterns of all three ZIKV genotypes was greater than the selection pressure imposed by *Ae. aegypti* or *H. sapiens*. This pattern of influence by *Ae. albopictus* remained consistent when measured in the viral genome as a whole and in individual coding sequences.

The sudden onset of increased and damaging interaction between ZIKV and humans is still contentious. The evidence of a link between ZIKV and neurological disorders has become stronger as increasingly convincing data continue to emerge. In fact, the association between ZIKV and neurological disorders dates back to the French Polynesian outbreak in 2013.^46^ The question that remains is what made ZIKV so lethal within such a short period of time? Whole-genome comparative analyses of the earlier and present endemic ZIKV strains have identified several mutations that might have changed ZIKV into a lethal virus,^47^ but confirmation of their relevance requires further investigation. RNA viruses are susceptible to mutation, but not every mutation observed makes the virus more virulent or its infection of its host more efficient. Instead, we suggest that in multiple-host multiple-vector viruses, the association between any new mutation and virulence must be examined with care because the effect of a new mutation could be the other way around, ie, virus acquiring new mutations as an efficient replication strategy for one specific host or vector and the associated increased virulence may come as a complementary offer for other. For instance, our SiD analysis suggested that the selection pressure induced by *Ae. albopictus* was strongest among those exerted by the *H. sapiens* and *Ae. aegypti*. It is quite possible that ZIKV was developing strong ties with *Ae. albopictus* and, during this chain of events, introduced new mutations within its genome that also became causative agents of neurological disorders in *H. sapiens*. The question arises: why was *Ae. albopictus* favored when *Ae. aegypti* was already present? We hypothesize that this is attributable to changes in the vector population itself. To test this hypothesis, we analyzed the recently reported global occurrence data for *Ae. aegypti* and *Ae. albopictus*^48^ and drew an interesting conclusion. A significant reduction in the population of *Ae. aegypti* and a sudden increase in the *Ae. albopictus* population was noted in Brazil and neighboring regions in 2014–2015, coincident with the emergence of the ZIKV epidemic. *Ae. albopictus* is an aggressive, strongly anthropophilic, exophagic, exophilic mosquito that has shown a dramatic expansion from its native tropical region of Southeast Asia to the temperate regions of Europe and North America in the last 30–40 years and is now found on every continent except Antarctica. Its successful adaptation to urban environments, diurnal feeding behavior, and habit of biting multiple hosts during the developmental cycle of its eggs has made *Ae. albopictus* very efficient at transmitting dengue fever, chikungunya disease and yellow fever.^49, 50, 51^ It is quite possible that the adaptation of ZIKV to *Ae. albopictus* will be enhanced even further in the future, beyond its current adaptation to *Ae. aegypti*, as the range of *Ae. albopictus* continues to increase. These observations of the evolution of ZIKV toward *Ae. albopictus* parallel the adaptive evolution of CHIKV toward *Ae. albopictus*, previously reported by us and other*s*.^34, 52^ Much convincing evidence has recently emerged supporting this notion, including: (i) both ZIKV and CHIKV have been repeatedly shown to follow similar patterns of evolution and spread in different regions;^53^ (ii) in an environment containing both these species of *Aedes* mosquitoes, significantly greater numbers of *Ae. albopictus* than *Ae. aegypti* were found to be ZIKV positive, and *Ae. albopictus* was identified as the primary vector for a ZIKV outbreak in Gabon;^54^ and (iii) significant increases in the geographic distributions of *Ae. albopictus* in Asia, Africa, Europe and the Americas were observed in a recent large-scale global distribution analysis.^48^

Presently, the major bottleneck limiting our complete understanding of the ongoing ZIKV outbreak is a lack of full ZIKV genome sequences, particularly those isolated from its vectors. It is not yet known how ZIKV responds to and manages multiple vectors which even though members of the same genus (*Aedes*) differ in certain genetic features and environmental preferences. Currently, no genomic sequence of ZIKV isolated from *Ae. aegypti* or *Ae. albopictus* populations in the affected areas have been reported. Therefore, at this stage, it is unclear whether either or both species of *Aedes* actually harbor ZIKV strains similar to those reported in the outbreak regions. When we looked at previously reported ZIKV strains, only a single strain was isolated from *Ae. aegypti*, in 1966. Although this strain currently clusters within the Asian-genotype group, our CA plots, for instance, demonstrate that this strain contains several differences at the level of individual genes from the rest of the Asian strains. Whether these genome-level differences are host- or vector-specific warrants further investigation. In the current scenario, the host–virus–vector triangle constitutes an important concern when reevaluating the current surveillance, eradication, and preventative strategies for ZIKV, which currently appear to focus on *Ae. aegypti*.

## Figures and Tables

**Table 1:** The relative synonymous codon usage (RSCU) patterns of ZIKV, its host and transmission vectors

		**ZIKV**				**Host and vectors**					**ZIKV**				**Host and vectors**		
**AA**	**Codon**	**Overall**	**Asian**	**EA**	**WA**	**HS**	**AG**	**AB**	**AA**	**Codon**	**Overall**	**Asian**	**EA**	**WA**	**HS**	**AG**	**AB**
Phe	UUU	0.96	0.92	**1.19**	0.93	0.92	0.56	0.48	Ser	UCU	0.66	0.64	0.79	0.57	1.14	0.66	0.54
	**UUC**	**1.04**	**1.08**	0.81	**1.07**	**1.08**	**1.44**	**1.52**		UCC	0.90	0.89	0.89	1.12	1.32	1.20	1.38
Leu	UUA	0.32	0.31	0.40	0.30	0.48	0.36	0.24		**UCA**	**1.40**	**1.43**	**1.35**	1.11	0.90	0.66	0.48
	UUG	1.32	1.31	1.37	1.36	0.78	1.32	1.14		UCG	0.60	0.58	0.61	0.74	0.30	**1.44**	**1.68**
	CUU	0.68	0.65	0.80	0.78	0.78	0.66	0.48		AGU	1.13	1.07	1.31	**1.34**	0.90	0.96	0.78
	CUC	1.00	1.01	0.99	0.92	1.20	0.84	0.84		AGC	1.32	1.39	1.05	1.12	**1.44**	1.08	1.08
	CUA	0.67	0.67	0.60	0.77	0.42	0.54	0.54	Arg	**AGA**	**2.64**	**2.66**	**2.61**	**2.49**	**1.26**	0.66	0.60
	**CUG**	**2.01**	**2.06**	**1.85**	**1.88**	**2.40**	**2.28**	**2.76**		CGU	0.41	0.41	0.39	0.41	0.48	**1.38**	**1.50**
Ile	AUU	0.88	0.89	0.93	0.78	1.08	0.99	0.75		CGC	0.33	0.32	0.37	0.38	1.08	1.26	1.32
	**AUC**	**1.13**	**1.09**	**1.22**	**1.26**	**1.41**	**1.59**	**1.86**		CGA	0.22	0.17	0.39	0.40	0.66	1.20	0.96
	AUA	1.00	1.02	0.86	0.96	0.51	0.39	0.39		CGG	0.78	0.81	0.70	0.66	1.20	1.02	1.20
Val	GUU	0.71	0.68	0.82	0.73	0.72	1.04	0.88		AGG	1.63	1.64	1.55	1.68	**1.26**	0.54	0.42
	GUC	1.20	1.27	0.95	1.04	0.96	1.08	**1.32**	Cys	**UGU**	**0.97**	**0.94**	**1.11**	**1.07**	0.92	0.84	0.70
	GUA	0.43	0.39	0.60	0.58	0.48	0.60	0.52		UGC	0.83	0.86	0.69	0.73	**1.08**	**1.16**	**1.30**
	**GUG**	**1.66**	**1.67**	**1.63**	**1.67**	**1.84**	**1.28**	**1.32**	His	CAU	0.81	0.80	0.80	**0.92**	0.84	0.84	0.76
Pro	CCU	0.62	0.63	0.67	0.52	1.16	0.68	0.36		**CAC**	**0.99**	**1.00**	**1.00**	0.89	**1.16**	**1.16**	**1.24**
	CCC	1.16	1.16	1.12	1.26	**1.28**	0.84	1.12	Gln	**CAA**	**0.94**	**0.96**	**0.89**	0.74	0.54	0.82	0.60
	**CCA**	**1.83**	**1.81**	**1.90**	**1.80**	1.12	1.20	1.08		CAG	0.67	0.64	0.71	**0.86**	**1.46**	**1.18**	**1.40**
	CCG	0.39	0.40	0.31	0.42	0.44	**1.32**	**1.44**	Asn	AAU	0.66	0.63	0.79	0.76	0.94	0.80	0.64
Thr	ACU	1.05	1.06	1.01	0.95	1.00	0.80	0.64		**AAC**	**1.34**	**1.37**	**1.21**	**1.24**	**1.06**	**1.20**	**1.36**
	ACC	1.04	1.04	1.03	1.00	**1.44**	**1.48**	**1.80**	Lys	AAA	0.75	0.76	0.70	0.76	0.86	0.80	0.58
	**ACA**	**1.49**	**1.44**	**1.66**	**1.73**	1.12	0.72	0.60		**AAG**	**1.25**	**1.24**	**1.30**	**1.24**	**1.14**	**1.20**	**1.42**
	ACG	0.43	0.46	0.31	0.32	0.44	1.00	1.00	Asp	GAU	0.95	0.97	0.76	0.92	0.92	**1.12**	0.96
Ala	GCU	1.10	1.10	1.08	1.14	1.08	1.08	1.00		**GAC**	**1.06**	**1.03**	**1.24**	**1.09**	**1.08**	0.88	**1.04**
	**GCC**	**1.26**	**1.25**	**1.30**	**1.29**	**1.60**	**1.48**	**1.80**	Glu	GAA	0.99	0.99	**1.01**	0.88	0.84	**1.16**	**1.10**
	GCA	1.15	1.12	1.27	1.23	0.92	0.76	0.60		**GAG**	**1.02**	**1.01**	0.99	**1.12**	**1.16**	0.84	0.90
	GCG	0.49	0.54	0.35	0.34	0.44	0.68	0.60	Gly	GGU	0.56	0.54	0.62	0.56	0.64	1.12	**1.24**
Tyr	UAU	0.48	0.44	0.61	0.58	0.88	0.64	0.56		GGC	0.67	0.69	0.61	0.66	**1.36**	1.04	1.08
	**UAC**	**1.12**	**1.16**	**0.99**	**1.02**	**1.12**	**1.36**	**1.44**		**GGA**	**1.74**	**1.70**	**1.93**	**1.82**	1.00	**1.48**	1.20
										GGG	1.03	1.07	0.83	0.97	1.00	0.36	0.48

Abbreviations: amino acid, AA; *H. sapiens*, HS; *Ae. aegypti*, AG; *Ae. Albopictus*, AB.

Preferred codons of ZIKV, *H. sapiens*, *Ae. aegypti* and *Ae. albopictus* are shown in bold.
