# Supplementary material for: Evolution of codon usage in Zika virus genomes is host and vector specific
Source: Emerg Microbes Infect. 2016 Oct 12;5(10):e107–. doi: 10.1038/emi.2016.106 (PMC5117728; doi:10.1038/emi.2016.106)
Supplement: Supplementary Figure S1 [file emi2016106x1.pdf]

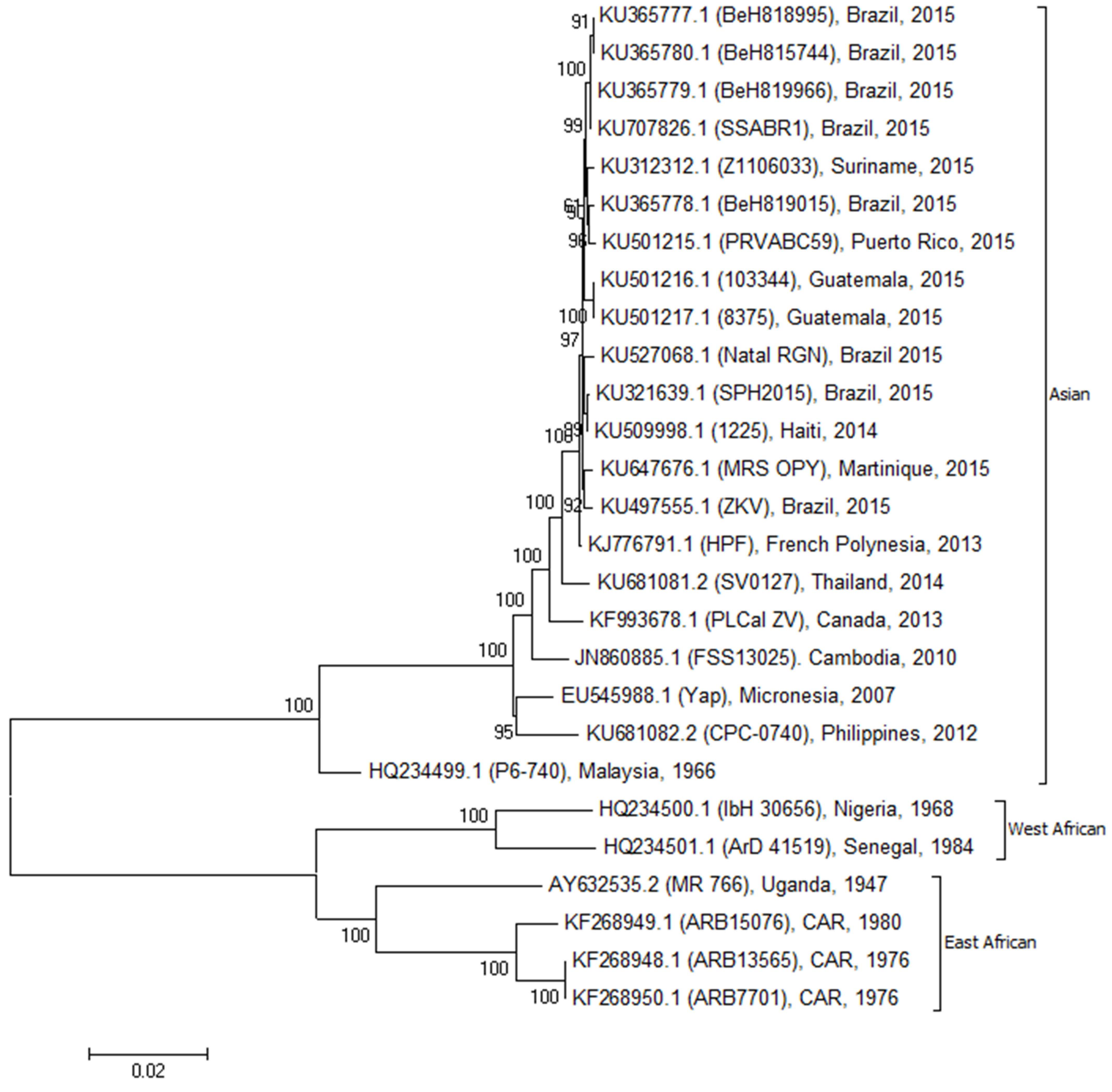

Supplementary Figure S1. Phylogenetic analysis of the ZIKV strains

The maximum likelihood tree was constructed from whole-genome sequences with the TN93+G model and 1000 bootstrap replicates. Scale bar indicates the number of nucleotide substitutions per site.
