# Supplementary material for: Evolution of codon usage in Zika virus genomes is host and vector specific
Source: Emerg Microbes Infect. 2016 Oct 12;5(10):e107–. doi: 10.1038/emi.2016.106 (PMC5117728; doi:10.1038/emi.2016.106)
Supplement: Supplementary Figure S3 [file emi2016106x3.pdf]

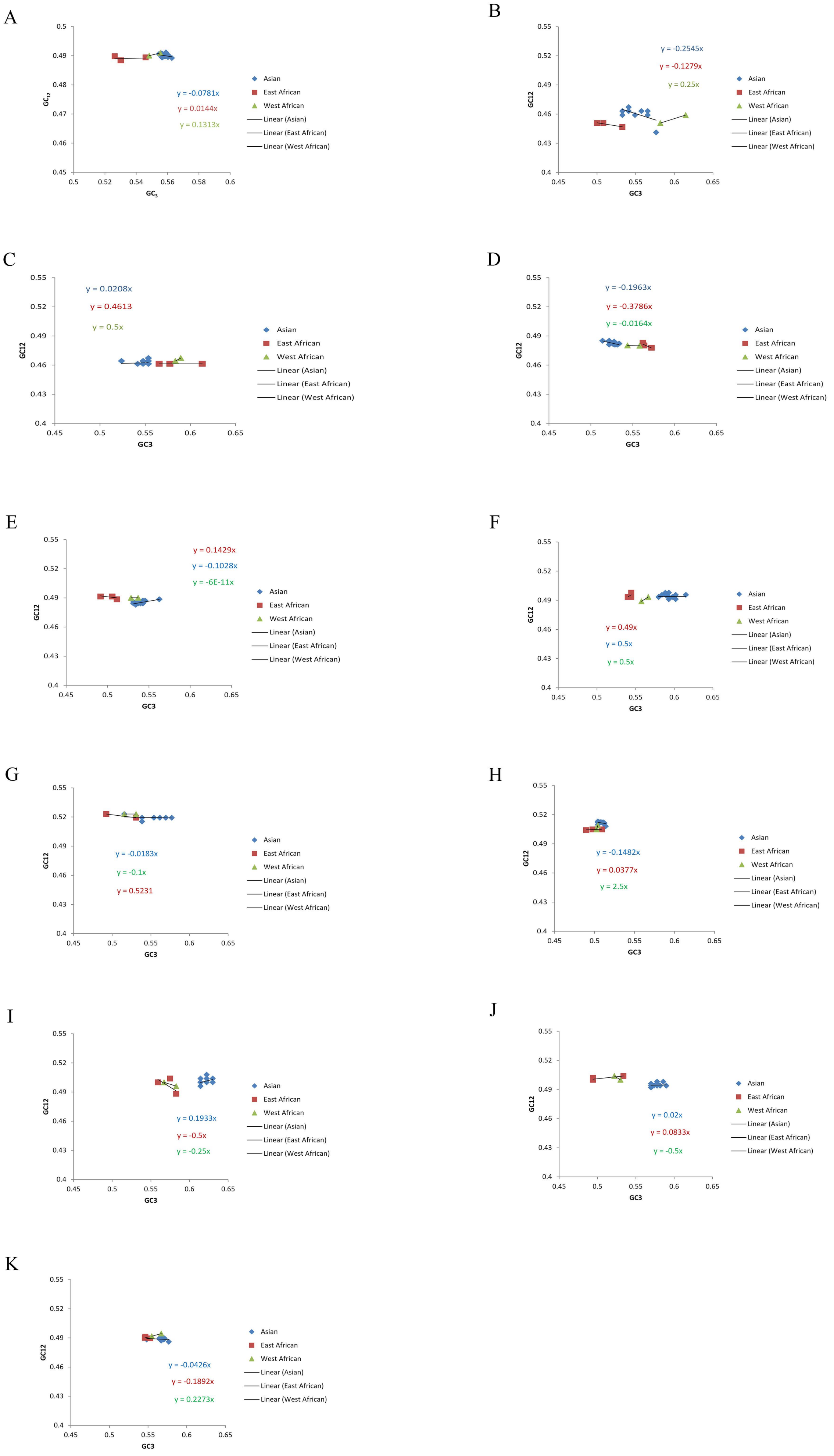

Supplementary Figure S3. Neutrality plot analysis.

Neutrality plots ($GC_{1,2}$ and that of the third codon position (GC_3)) were constructed for whole genome and individual ZIKV coding sequences. (A) Whole genome. (B) *C*. (C) *prM*. (D) *E*. (E) *NS1*. (F) *NS2A*. (G) *NS2B*. (H) *NS3*. (I) *NS4A*. (J) *NS4B*. (K) *NS5*.
