# Supplementary material for: Evolution of codon usage in Zika virus genomes is host and vector specific
Source: Emerg Microbes Infect. 2016 Oct 12;5(10):e107–. doi: 10.1038/emi.2016.106 (PMC5117728; doi:10.1038/emi.2016.106)
Supplement: Supplementary Table S1 [file emi2016106x4.pdf]

Supplementary Table S1: Demographics of ZIKV genomes analyzed in present study.

S. No	GenBank Accession	Year	Host	Country	Genotype[1]	
1	KF268948	1976	*Aedes africanus*	Central African Republic	East African	
2	EU545988	2007	*Homo sapiens*	Micronesia	Asian	
3	KU681082	2012	*Homo sapiens*	Philippines	Asian	
4	KJ776791	2013	*Homo sapiens*	French Polynesia	Asian	
5	KU681081	2014	*Homo sapiens*	Thailand	Asian	
6	KU509998	2014	*Homo sapiens*	Haiti	Asian	
7	KU365779	2015	*Homo sapiens*	Brazil	Asian	
8	KU497555	2015	*Homo sapiens*	Brazil	Asian	
9	KU647676	2015	*Homo sapiens*	Martinique	Asian	
10	KU527068	2015	*Homo sapiens*	Brazil	Asian	
11	KU707826	2015	*Homo sapiens*	Brazil	Asian	
12	KU501215	2015	*Homo sapiens*	Puerto Rico	Asian	
13	KU501216	2015	*Homo sapiens*	Guatemala	Asian	
14	KU501217	2015	*Homo sapiens*	Guatemala	Asian	
15	KU365780	2015	*Homo sapiens*	Brazil	Asian	
16	KU365778	2015	*Homo sapiens*	Brazil	Asian	
17	KU312312	2015	*Homo sapiens*	Suriname	Asian	
18	KU365777	2015	*Homo sapiens*	Brazil	Asian	
19	KU321639	2015	*Homo sapiens*	Brazil	Asian	
20	AY632535	1947	*Sentinel monkey*	Uganda	East African	
21	KF268949	1980	*Aedes opok*	Central African Republic	East African	
22	KF268950	1976	*Aedes africanus*	Central African Republic	East African	
23	HQ234501	1984	*Aedes africanus*	Senegal	West African	
24	HQ234500	1968	*Homo sapiens*	Nigeria	West African	
25	KF993678	2013	*Homo sapiens*	Canada	Asian	
26	HQ234499	1966	*Aedes Aegypti*	Malaysia	Asian	
27	JN860885	2010	*Homo sapiens*	Cambodia	Asian	
28	KF383116	1968	*Aedes luteocephalus*	Senegal	East African	Recombinants
29	KF383117	1997	*Aedes luteocephalus*	Senegal	East African	Recombinants
30	KF383118	2001	*Aedes africanus*	Senegal	East African	Recombinants
31	KF383119	2001	*Aedes africanus*	Senegal	East African	Recombinants

[1]; inferred from the phylogenetic analysis (Supplementary Figure S1)
