# Supplementary material for: Evolution of codon usage in Zika virus genomes is host and vector specific
Source: Emerg Microbes Infect. 2016 Oct 12;5(10):e107–. doi: 10.1038/emi.2016.106 (PMC5117728; doi:10.1038/emi.2016.106)
Supplement: Supplementary Table S2 [file emi2016106x5.pdf]

Supplementary Table S2: Nucleotide composition analysis of ZIKV coding sequences (%).

No	A	U	G	C	A_3	U_3	G_3	C_3	AU	GC	GC_1	GC_2	AU_3	GC_3	GC_{12}	ENC
1	27.10	22.66	28.82	21.42	26.20	20.76	29.05	23.99	49.76	50.24	53.04	44.65	46.96	53.04	48.84	54.03
2	26.52	22.12	29.39	21.97	24.40	19.57	30.89	25.15	48.64	51.36	53.05	44.99	43.96	56.04	49.02	54.28
3	26.76	22.00	29.22	22.02	25.13	19.23	30.43	25.21	48.76	51.24	53.04	45.05	44.36	55.64	49.05	54.04
4	26.67	22.04	29.28	22.00	24.84	19.28	30.64	25.24	48.72	51.28	52.94	45.03	44.12	55.88	48.98	53.75
5	26.70	22.03	29.20	22.07	25.06	19.30	30.34	25.30	48.73	51.27	53.12	45.05	44.36	55.64	49.08	54.14
6	26.66	22.04	29.28	22.01	24.82	19.29	30.62	25.27	48.71	51.29	52.96	45.03	44.11	55.89	48.99	53.60
7	26.70	22.04	29.25	22.02	24.92	19.27	30.52	25.29	48.74	51.26	52.95	45.03	44.19	55.81	48.99	53.64
8	26.70	21.99	29.24	22.07	24.93	19.04	30.52	25.51	48.69	51.31	52.88	45.03	43.97	56.03	48.95	53.52
9	26.74	22.02	29.23	22.01	25.07	19.21	30.46	25.27	48.77	51.23	52.91	45.07	44.28	55.72	48.99	53.50
10	26.69	22.06	29.25	22.00	24.99	19.23	30.46	25.32	48.75	51.25	52.99	44.99	44.22	55.78	48.99	53.63
11	26.70	22.04	29.25	22.02	24.92	19.27	30.52	25.29	48.74	51.26	52.95	45.03	44.19	55.81	48.99	53.64
12	26.72	21.92	29.25	22.12	24.98	18.98	30.52	25.53	48.64	51.36	52.95	45.10	43.95	56.05	49.02	53.82
13	26.73	22.09	29.19	21.99	25.03	19.33	30.39	25.25	48.82	51.18	52.85	45.04	44.36	55.64	48.95	53.95
14	26.73	22.08	29.19	22.00	25.03	19.29	30.39	25.29	48.81	51.19	52.85	45.04	44.32	55.68	48.95	53.89
15	26.69	22.05	29.25	22.00	24.92	19.31	30.52	25.25	48.75	51.25	52.96	45.03	44.22	55.78	48.99	53.64
16	26.74	22.01	29.21	22.04	25.04	19.20	30.40	25.37	48.75	51.25	52.96	45.03	44.23	55.77	48.99	53.85
17	26.68	21.99	29.27	22.07	24.87	19.14	30.57	25.42	48.66	51.34	52.94	45.09	44.01	55.99	49.01	53.64
18	26.69	22.05	29.25	22.01	24.92	19.29	30.52	25.27	48.74	51.26	52.96	45.03	44.21	55.79	48.99	53.64
19	26.65	22.04	29.30	22.01	24.75	19.29	30.69	25.27	48.69	51.31	52.94	45.03	44.04	55.96	48.98	53.55
20	27.05	22.12	29.01	21.82	25.93	19.47	29.66	24.94	49.17	50.83	53.23	44.66	45.40	54.60	48.95	54.71
21	27.20	22.60	28.77	21.42	26.56	20.81	28.90	23.72	49.80	50.20	53.19	44.78	47.38	52.62	48.98	54.29
22	27.10	22.67	28.81	21.42	26.20	20.81	29.00	23.99	49.77	50.23	53.04	44.65	47.00	53.00	48.84	54.23
23	26.75	22.00	29.25	21.99	25.41	19.06	30.20	25.33	48.79	51.24	53.30	44.90	44.47	55.53	49.10	54.28
24	26.90	22.16	29.24	21.70	25.63	19.54	30.22	24.61	49.02	50.95	53.26	44.74	45.17	54.83	49.00	54.10
25	26.69	21.93	29.27	22.10	24.68	19.05	30.91	25.37	48.63	51.37	52.92	44.92	43.73	56.27	48.92	53.32
26	26.53	22.10	29.35	22.02	24.62	19.48	30.63	25.27	48.62	51.38	53.19	45.05	44.10	55.90	49.12	54.90
27	26.66	22.15	29.27	21.92	24.74	19.81	30.65	24.80	48.81	51.19	53.08	45.04	44.55	55.45	49.06	54.43
Avg	26.76	22.11	29.20	21.94	25.13	19.46	30.32	25.09	48.87	51.13	53.02	44.96	44.59	55.41	48.99	53.93
SD	0.17	0.20	0.16	0.20	0.52	0.51	0.53	0.47	0.35	0.35	0.12	0.14	0.97	0.97	0.06	0.39

ENC effective number of codons; *Avg* average; *SD* standard deviation. The numbering of ZIKV strains in first column corresponds to that of Supplementary Table S1.
