# Supplementary material for: Evolution of codon usage in Zika virus genomes is host and vector specific
Source: Emerg Microbes Infect. 2016 Oct 12;5(10):e107–. doi: 10.1038/emi.2016.106 (PMC5117728; doi:10.1038/emi.2016.106)
Supplement: Supplementary Table S3 [file emi2016106x6.pdf]

Supplementary Table S3. Summary of correlation between the first two principle axes and nucleotide constraints in ZIKV (average of all genes)

**Base composition**	**f'$_1$ (Axis 1)**	**f'$_2$ (Axis 2)**
A	0.449*	-0.029 ^{NS}
T	0.436*	0.134 ^{NS}
G	-0.224 ^{NS}	-0.057 ^{NS}
C	-0.526**	0.075 ^{NS}
A_3	0.441*	-0.004 ^{NS}
T_3	0.491**	0.093 ^{NS}
G_3	-0.438*	-0.026 ^{NS}
C_3	-0.545**	0.025 ^{NS}
AU	0.425*	0.055 ^{NS}
GC	-0.420*	-0.061 ^{NS}
AU_3	0.552**	0.109 ^{NS}
GC_3	-0.552**	-0.109 ^{NS}

NS means non-significant ($P > 0.05$).

* represents $P < 0.05$.

** represents $P < 0.01$.
