# Supplementary material for: Evolution of codon usage in Zika virus genomes is host and vector specific
Source: Emerg Microbes Infect. 2016 Oct 12;5(10):e107–. doi: 10.1038/emi.2016.106 (PMC5117728; doi:10.1038/emi.2016.106)
Supplement: Supplementary Table S4 [file emi2016106x7.pdf]

Supplementary Table S4. Summary of codon adaptation index (CAI) analysis.

**ZIKV Coding Sequences**			***C***			***E***			***NS1***			***NS2A***			***NS2B***	
**ZIKV Strains**		**HS**	**AG**	**AB**	**HS**	**AG**	**AB**	**HS**	**AG**	**AB**	**HS**	**AG**	**AB**	**HS**	**AG**	**AB**
KF268948|ARB13565	**EA**	0.76	0.73	0.67	0.78	0.74	0.68	0.77	0.73	0.67	0.75	0.71	0.64	0.75	0.72	0.65
EU545988|FSM	**Asian**	0.76	0.73	0.67	0.75	0.72	0.65	0.78	0.74	0.68	0.74	0.73	0.66	0.72	0.72	0.67
KU681082|CPC-0740	**Asian**	0.76	0.72	0.66	0.75	0.72	0.65	0.79	0.75	0.69	0.75	0.73	0.65	0.72	0.71	0.66
KJ776791|H/PF/2013	**Asian**	0.76	0.73	0.66	0.76	0.73	0.66	0.78	0.75	0.68	0.75	0.74	0.67	0.72	0.72	0.68
KU681081|SV0127/14	**Asian**	0.76	0.74	0.67	0.76	0.73	0.66	0.78	0.75	0.69	0.75	0.73	0.65	0.72	0.71	0.66
KU509998|Haiti/1225/2014	**Asian**	0.76	0.73	0.66	0.76	0.72	0.65	0.79	0.75	0.69	0.75	0.73	0.66	0.72	0.72	0.68
KU365779|BeH819966	**Asian**	0.76	0.73	0.66	0.76	0.72	0.65	0.79	0.75	0.69	0.75	0.73	0.66	0.72	0.72	0.68
KU497555|Brazil-ZKV2015	**Asian**	0.76	0.74	0.67	0.76	0.72	0.65	0.78	0.74	0.68	0.75	0.73	0.66	0.73	0.72	0.68
KU647676|MRS_OPY	**Asian**	0.76	0.73	0.66	0.76	0.73	0.66	0.78	0.74	0.68	0.75	0.73	0.66	0.72	0.71	0.67
KU527068|Natal_RGN	**Asian**	0.76	0.73	0.66	0.76	0.73	0.66	0.78	0.74	0.69	0.75	0.73	0.66	0.72	0.72	0.68
KU707826|SSABR1	**Asian**	0.76	0.73	0.66	0.76	0.72	0.65	0.79	0.75	0.69	0.75	0.73	0.66	0.72	0.72	0.68
KU501215|PRVABC59	**Asian**	0.76	0.74	0.67	0.76	0.72	0.65	0.79	0.74	0.68	0.75	0.74	0.66	0.72	0.72	0.68
KU501216|103344	**Asian**	0.76	0.73	0.66	0.76	0.73	0.66	0.79	0.75	0.68	0.74	0.72	0.65	0.72	0.72	0.68
KU501217|8375	**Asian**	0.76	0.73	0.66	0.76	0.73	0.66	0.79	0.75	0.68	0.74	0.72	0.65	0.72	0.72	0.68
KU365780|BeH815744	**Asian**	0.76	0.73	0.66	0.76	0.72	0.65	0.79	0.75	0.69	0.75	0.73	0.66	0.72	0.72	0.68
KU365778|BeH819015	**Asian**	0.75	0.73	0.66	0.76	0.72	0.65	0.79	0.75	0.69	0.75	0.73	0.66	0.72	0.72	0.67
KU312312|Z1106033	**Asian**	0.76	0.73	0.66	0.76	0.72	0.65	0.78	0.75	0.69	0.75	0.73	0.66	0.72	0.72	0.68
KU365777|BeH818995	**Asian**	0.76	0.73	0.66	0.76	0.72	0.65	0.79	0.75	0.69	0.75	0.73	0.66	0.72	0.72	0.68
KU321639|ZikaSPH2015	**Asian**	0.77	0.73	0.67	0.76	0.72	0.65	0.79	0.75	0.69	0.75	0.73	0.66	0.72	0.72	0.68
AY632535|MR_766	**EA**	0.76	0.73	0.67	0.76	0.74	0.68	0.77	0.73	0.66	0.73	0.70	0.63	0.74	0.71	0.66
KF268949|ARB15076	**EA**	0.76	0.74	0.67	0.78	0.74	0.68	0.76	0.73	0.66	0.73	0.71	0.63	0.74	0.69	0.63
KF268950|ARB7701	**EA**	0.76	0.73	0.67	0.78	0.74	0.68	0.77	0.73	0.67	0.75	0.71	0.63	0.75	0.72	0.65
HQ234501|ArD_41519	**WA**	0.77	0.76	0.71	0.77	0.73	0.67	0.77	0.74	0.68	0.75	0.72	0.65	0.73	0.71	0.65
HQ234500|IbH_30656	**WA**	0.76	0.76	0.70	0.78	0.75	0.68	0.77	0.73	0.67	0.74	0.72	0.64	0.72	0.68	0.63
KF993678|PLCal_ZV	**Asian**	0.77	0.73	0.67	0.76	0.73	0.65	0.79	0.75	0.69	0.75	0.73	0.66	0.72	0.72	0.67
HQ234499|P6-740	**Asian**	0.75	0.74	0.68	0.75	0.73	0.66	0.77	0.75	0.69	0.75	0.73	0.66	0.74	0.71	0.66
JN860885|FSS13025	**Asian**	0.76	0.73	0.67	0.76	0.73	0.66	0.79	0.75	0.68	0.74	0.72	0.65	0.72	0.71	0.66
**Average**		0.76	0.73	0.67	0.76	0.73	0.66	0.78	0.74	0.68	0.74	0.73	0.65	0.73	0.71	0.67
**Standard deviation**		0.00	0.01	0.01	0.01	0.01	0.01	0.01	0.01	0.01	0.01	0.01	0.01	0.01	0.01	0.01

**ZIKV Coding Sequences**			***NS3***			***NS4A***			***NS4B***			***NS5***			***prM***				**Average (ALL)**	
**ZIKV Strains**		**HS**	**AG**	**AB**	**HS**	**AG**	**AB**	**HS**	**AG**	**AB**	**HS**	**AG**	**AB**	**HS**	**AG**	**AB**		**HS**	**AG**	**AB**
KF268948|ARB13565	**EA**	0.75	0.73	0.66	0.72	0.69	0.63	0.73	0.70	0.62	0.78	0.76	0.69	0.75	0.76	0.68		0.75	0.73	0.66
EU545988|FSM	**Asian**	0.74	0.71	0.64	0.74	0.73	0.67	0.74	0.71	0.65	0.77	0.74	0.68	0.76	0.74	0.67		0.75	0.73	0.66
KU681082|CPC-0740	**Asian**	0.75	0.71	0.64	0.74	0.72	0.65	0.75	0.71	0.64	0.76	0.74	0.68	0.74	0.73	0.65		0.75	0.72	0.66
KJ776791|H/PF/2013	**Asian**	0.75	0.71	0.64	0.74	0.72	0.65	0.74	0.71	0.64	0.77	0.74	0.68	0.75	0.74	0.66		0.75	0.73	0.66
KU681081|SV0127/14	**Asian**	0.75	0.71	0.65	0.73	0.72	0.66	0.74	0.71	0.65	0.77	0.74	0.68	0.75	0.74	0.66		0.75	0.73	0.66
KU509998|Haiti/1225/2014	**Asian**	0.75	0.71	0.64	0.74	0.72	0.65	0.75	0.72	0.65	0.77	0.74	0.68	0.75	0.74	0.66		0.75	0.73	0.66
KU365779|BeH819966	**Asian**	0.75	0.71	0.64	0.74	0.72	0.65	0.74	0.71	0.64	0.76	0.74	0.68	0.75	0.74	0.66		0.75	0.73	0.66
KU497555|Brazil-ZKV2015	**Asian**	0.75	0.71	0.64	0.74	0.72	0.65	0.74	0.71	0.64	0.77	0.74	0.68	0.75	0.74	0.66		0.75	0.73	0.66
KU647676|MRS_OPY	**Asian**	0.75	0.71	0.64	0.74	0.72	0.65	0.75	0.71	0.64	0.77	0.74	0.68	0.75	0.74	0.66		0.75	0.73	0.66
KU527068|Natal_RGN	**Asian**	0.75	0.71	0.64	0.74	0.72	0.65	0.74	0.71	0.64	0.77	0.74	0.68	0.75	0.74	0.66		0.75	0.73	0.66
KU707826|SSABR1	**Asian**	0.75	0.71	0.64	0.74	0.72	0.65	0.74	0.71	0.64	0.76	0.74	0.68	0.75	0.74	0.66		0.75	0.73	0.66
KU501215|PRVABC59	**Asian**	0.75	0.71	0.65	0.73	0.72	0.65	0.75	0.71	0.64	0.76	0.74	0.68	0.75	0.74	0.66		0.75	0.73	0.66
KU501216|103344	**Asian**	0.75	0.71	0.64	0.73	0.72	0.65	0.74	0.71	0.64	0.77	0.74	0.68	0.75	0.74	0.66		0.75	0.73	0.66
KU501217|8375	**Asian**	0.75	0.71	0.64	0.73	0.72	0.65	0.74	0.71	0.64	0.77	0.74	0.68	0.75	0.74	0.66		0.75	0.73	0.66
KU365780|BeH815744	**Asian**	0.75	0.71	0.64	0.74	0.72	0.65	0.74	0.71	0.64	0.76	0.74	0.68	0.75	0.73	0.65		0.75	0.73	0.66
KU365778|BeH819015	**Asian**	0.75	0.71	0.64	0.73	0.72	0.65	0.75	0.71	0.64	0.77	0.74	0.68	0.75	0.74	0.66		0.75	0.73	0.66
KU312312|Z1106033	**Asian**	0.75	0.71	0.64	0.74	0.72	0.66	0.75	0.71	0.64	0.76	0.74	0.68	0.75	0.74	0.66		0.75	0.73	0.66
KU365777|BeH818995	**Asian**	0.75	0.71	0.64	0.74	0.72	0.65	0.74	0.71	0.64	0.76	0.74	0.68	0.75	0.73	0.65		0.75	0.73	0.66
KU321639|ZikaSPH2015	**Asian**	0.75	0.71	0.64	0.74	0.72	0.65	0.75	0.72	0.65	0.77	0.74	0.68	0.75	0.74	0.66		0.75	0.73	0.66
AY632535|MR_766	**EA**	0.76	0.73	0.66	0.73	0.69	0.62	0.74	0.71	0.63	0.78	0.76	0.69	0.76	0.76	0.69		0.75	0.73	0.66
KF268949|ARB15076	**EA**	0.76	0.73	0.66	0.70	0.68	0.61	0.73	0.70	0.62	0.78	0.76	0.70	0.75	0.76	0.68		0.75	0.72	0.65
KF268950|ARB7701	**EA**	0.75	0.73	0.66	0.72	0.69	0.63	0.73	0.70	0.62	0.78	0.76	0.69	0.75	0.76	0.68		0.75	0.73	0.66
HQ234501|ArD_41519	**WA**	0.75	0.72	0.65	0.73	0.71	0.64	0.76	0.71	0.63	0.78	0.76	0.69	0.72	0.73	0.67		0.75	0.73	0.67
HQ234500|IbH_30656	**WA**	0.75	0.72	0.65	0.70	0.69	0.62	0.76	0.72	0.64	0.78	0.76	0.70	0.75	0.74	0.67		0.75	0.73	0.66
KF993678|PLCal_ZV	**Asian**	0.75	0.72	0.65	0.74	0.71	0.65	0.75	0.71	0.64	0.77	0.74	0.68	0.75	0.73	0.66		0.75	0.73	0.66
HQ234499|P6-740	**Asian**	0.75	0.72	0.65	0.75	0.71	0.65	0.74	0.71	0.63	0.77	0.74	0.68	0.76	0.74	0.66		0.75	0.73	0.66
JN860885|FSS13025	**Asian**	0.75	0.72	0.64	0.73	0.70	0.64	0.74	0.71	0.64	0.76	0.74	0.68	0.76	0.74	0.66		0.75	0.72	0.66
**Average**		0.75	0.72	0.65	0.73	0.71	0.64	0.74	0.71	0.64	0.77	0.75	0.68	0.75	0.74	0.66		0.75	0.73	0.66
**Standard deviation**		0.00	0.01	0.01	0.01	0.01	0.01	0.01	0.00	0.01	0.01	0.01	0.01	0.01	0.01	0.01				
																	**HS**	**0.78**	**NS1**	
																	**AG**	**0.75**	**NS5**	
																	AB	***0.68***	***NS5***	

**ZIKV Coding Sequences**			***C***			***E***			***NS1***			***NS2A***			***NS2B***	
**ZIKV Strains (Asian)**		**HS**	**AG**	**AB**	**HS**	**AG**	**AB**	**HS**	**AG**	**AB**	**HS**	**AG**	**AB**	**HS**	**AG**	**AB**
gb:EU545988		0.76	0.73	0.67	0.75	0.72	0.65	0.78	0.74	0.68	0.74	0.73	0.66	0.72	0.72	0.67
gb:KU681082		0.76	0.72	0.66	0.75	0.72	0.65	0.79	0.75	0.69	0.75	0.73	0.65	0.72	0.71	0.66
gb:KJ776791		0.76	0.73	0.66	0.76	0.73	0.66	0.78	0.75	0.68	0.75	0.74	0.67	0.72	0.72	0.68
gb:KU681081		0.76	0.74	0.67	0.76	0.73	0.66	0.78	0.75	0.69	0.75	0.73	0.65	0.72	0.71	0.66
gb:KU509998		0.76	0.73	0.66	0.76	0.72	0.65	0.79	0.75	0.69	0.75	0.73	0.66	0.72	0.72	0.68
gb:KU365779		0.76	0.73	0.66	0.76	0.72	0.65	0.79	0.75	0.69	0.75	0.73	0.66	0.72	0.72	0.68
gb:KU497555		0.76	0.74	0.67	0.76	0.72	0.65	0.78	0.74	0.68	0.75	0.73	0.66	0.73	0.72	0.68
gb:KU647676		0.76	0.73	0.66	0.76	0.73	0.66	0.78	0.74	0.68	0.75	0.73	0.66	0.72	0.71	0.67
gb:KU527068		0.76	0.73	0.66	0.76	0.73	0.66	0.78	0.74	0.69	0.75	0.73	0.66	0.72	0.72	0.68
gb:KU707826		0.76	0.73	0.66	0.76	0.72	0.65	0.79	0.75	0.69	0.75	0.73	0.66	0.72	0.72	0.68
gb:KU501215		0.76	0.74	0.67	0.76	0.72	0.65	0.79	0.74	0.68	0.75	0.74	0.66	0.72	0.72	0.68
gb:KU501216		0.76	0.73	0.66	0.76	0.73	0.66	0.79	0.75	0.68	0.74	0.72	0.65	0.72	0.72	0.68
gb:KU501217		0.76	0.73	0.66	0.76	0.73	0.66	0.79	0.75	0.68	0.74	0.72	0.65	0.72	0.72	0.68
gb:KU365780		0.76	0.73	0.66	0.76	0.72	0.65	0.79	0.75	0.69	0.75	0.73	0.66	0.72	0.72	0.68
gb:KU365778		0.75	0.73	0.66	0.76	0.72	0.65	0.79	0.75	0.69	0.75	0.73	0.66	0.72	0.72	0.67
gb:KU312312		0.76	0.73	0.66	0.76	0.72	0.65	0.78	0.75	0.69	0.75	0.73	0.66	0.72	0.72	0.68
gb:KU365777		0.76	0.73	0.66	0.76	0.72	0.65	0.79	0.75	0.69	0.75	0.73	0.66	0.72	0.72	0.68
gb:KU321639		0.77	0.73	0.67	0.76	0.72	0.65	0.79	0.75	0.69	0.75	0.73	0.66	0.72	0.72	0.68
gb:KF993678		0.77	0.73	0.67	0.76	0.73	0.65	0.79	0.75	0.69	0.75	0.73	0.66	0.72	0.72	0.67
gb:HQ234499		0.75	0.74	0.68	0.75	0.73	0.66	0.77	0.75	0.69	0.75	0.73	0.66	0.74	0.71	0.66
gb:JN860885		0.76	0.73	0.67	0.76	0.73	0.66	0.79	0.75	0.68	0.74	0.72	0.65	0.72	0.71	0.66

**Average**		0.76	0.73	0.67		0.76	0.72	0.65		0.78	0.75	0.68		0.75	0.73	0.66		0.72	0.72	0.67				
**Standar deviation**		0.00	0.00	0.00		0.00	0.00	0.00		0.00	0.00	0.00		0.00	0.00	0.00		0.00	0.00	0.01				
**ZIKV Coding Sequences**			***NS3***				***NS4A***				***NS4B***				***NS5***				***prM***				**Average (ALL)**	
**ZIKV Strains (Asian)**		**HS**	**AG**	**AB**		**HS**	**AG**	**AB**		**HS**	**AG**	**AB**		**HS**	**AG**	**AB**		**HS**	**AG**	**AB**		**HS**	**AG**	**AB**
gb:EU545988		0.74	0.71	0.64		0.74	0.73	0.67		0.74	0.71	0.65		0.77	0.74	0.68		0.76	0.74	0.67		0.75	0.73	0.66
gb:KU681082		0.75	0.71	0.64		0.74	0.72	0.65		0.75	0.71	0.64		0.76	0.74	0.68		0.74	0.73	0.65		0.75	0.72	0.66
gb:KJ776791		0.75	0.71	0.64		0.74	0.72	0.65		0.74	0.71	0.64		0.77	0.74	0.68		0.75	0.74	0.66		0.75	0.73	0.66
gb:KU681081		0.75	0.71	0.65		0.73	0.72	0.66		0.74	0.71	0.65		0.77	0.74	0.68		0.75	0.74	0.66		0.75	0.73	0.66
gb:KU509998		0.75	0.71	0.64		0.74	0.72	0.65		0.75	0.72	0.65		0.77	0.74	0.68		0.75	0.74	0.66		0.75	0.73	0.66
gb:KU365779		0.75	0.71	0.64		0.74	0.72	0.65		0.74	0.71	0.64		0.76	0.74	0.68		0.75	0.74	0.66		0.75	0.73	0.66
gb:KU497555		0.75	0.71	0.64		0.74	0.72	0.65		0.74	0.71	0.64		0.77	0.74	0.68		0.75	0.74	0.66		0.75	0.73	0.66
gb:KU647676		0.75	0.71	0.64		0.74	0.72	0.65		0.75	0.71	0.64		0.77	0.74	0.68		0.75	0.74	0.66		0.75	0.73	0.66
gb:KU527068		0.75	0.71	0.64		0.74	0.72	0.65		0.74	0.71	0.64		0.77	0.74	0.68		0.75	0.74	0.66		0.75	0.73	0.66
gb:KU707826		0.75	0.71	0.64		0.74	0.72	0.65		0.74	0.71	0.64		0.76	0.74	0.68		0.75	0.74	0.66		0.75	0.73	0.66
gb:KU501215		0.75	0.71	0.65		0.73	0.72	0.65		0.75	0.71	0.64		0.76	0.74	0.68		0.75	0.74	0.66		0.75	0.73	0.66
gb:KU501216		0.75	0.71	0.64		0.73	0.72	0.65		0.74	0.71	0.64		0.77	0.74	0.68		0.75	0.74	0.66		0.75	0.73	0.66
gb:KU501217		0.75	0.71	0.64		0.73	0.72	0.65		0.74	0.71	0.64		0.77	0.74	0.68		0.75	0.74	0.66		0.75	0.73	0.66
gb:KU365780		0.75	0.71	0.64		0.74	0.72	0.65		0.74	0.71	0.64		0.76	0.74	0.68		0.75	0.73	0.65		0.75	0.73	0.66
gb:KU365778		0.75	0.71	0.64		0.73	0.72	0.65		0.75	0.71	0.64		0.77	0.74	0.68		0.75	0.74	0.66		0.75	0.73	0.66
gb:KU312312		0.75	0.71	0.64		0.74	0.72	0.66		0.75	0.71	0.64		0.76	0.74	0.68		0.75	0.74	0.66		0.75	0.73	0.66
gb:KU365777		0.75	0.71	0.64		0.74	0.72	0.65		0.74	0.71	0.64		0.76	0.74	0.68		0.75	0.73	0.65		0.75	0.73	0.66
gb:KU321639		0.75	0.71	0.64		0.74	0.72	0.65		0.75	0.72	0.65		0.77	0.74	0.68		0.75	0.74	0.66		0.75	0.73	0.66
gb:KF993678		0.75	0.72	0.65		0.74	0.71	0.65		0.75	0.71	0.64		0.77	0.74	0.68		0.75	0.73	0.66		0.75	0.73	0.66
gb:HQ234499		0.75	0.72	0.65		0.75	0.71	0.65		0.74	0.71	0.63		0.77	0.74	0.68		0.76	0.74	0.66		0.75	0.73	0.66
gb:JN860885		0.75	0.72	0.64		0.73	0.70	0.64		0.74	0.71	0.64		0.76	0.74	0.68		0.76	0.74	0.66		0.75	0.72	0.66
**Average**		0.75	0.71	0.64		0.74	0.72	0.65		0.74	0.71	0.64		0.77	0.74	0.68		0.75	0.74	0.66		0.75	0.73	0.66
**Standar deviation**		0.00	0.00	0.00		0.00	0.00	0.00		0.00	0.00	0.00		0.00	0.00	0.00		0.00	0.00	0.00		0.00	0.00	0.00
																					**HS**	**0.78**	**NS1**	
																					**AG**	**0.75**	**NS1**	
																					**AB**	**0.68**	**NS1**	
**ZIKV Coding Sequences**			***C***				***E***				***NS1***				***NS2A***				***NS2B***					
**ZIKV Strains (EA)**		**HS**	**AG**	**AB**		**HS**	**AG**	**AB**		**HS**	**AG**	**AB**		**HS**	**AG**	**AB**		**HS**	**AG**	**AB**				
gb:KF268948		0.76	0.73	0.67		0.78	0.74	0.68		0.77	0.73	0.67		0.75	0.71	0.64		0.75	0.72	0.65				
gb:AY632535		0.76	0.73	0.67		0.76	0.74	0.68		0.77	0.73	0.66		0.73	0.70	0.63		0.74	0.71	0.66				
gb:KF268949		0.76	0.74	0.67		0.78	0.74	0.68		0.76	0.73	0.66		0.73	0.71	0.63		0.74	0.69	0.63				
gb:KF268950		0.76	0.73	0.67		0.78	0.74	0.68		0.77	0.73	0.67		0.75	0.71	0.63		0.75	0.72	0.65				
**Average**		0.76	0.73	0.67		0.77	0.74	0.68		0.77	0.73	0.66		0.74	0.71	0.63		0.74	0.71	0.65				
**Standard deviation**		0.00	0.00	0.00		0.01	0.00	0.00		0.00	0.00	0.00		0.01	0.00	0.00		0.01	0.01	0.01				
**ZIKV Coding Sequences**			***NS3***				***NS4A***				***NS4B***				***NS5***				***prM***				**Average (ALL)**	
**ZIKV Strains (EA)**		**HS**	**AG**	**AB**		**HS**	**AG**	**AB**		**HS**	**AG**	**AB**		**HS**	**AG**	**AB**		**HS**	**AG**	**AB**		**HS**	**AG**	**AB**
gb:KF268948		0.75	0.73	0.66		0.72	0.69	0.63		0.73	0.70	0.62		0.78	0.76	0.69		0.75	0.76	0.68		0.75	0.73	0.66
gb:AY632535		0.76	0.73	0.66		0.73	0.69	0.62		0.74	0.71	0.63		0.78	0.76	0.69		0.76	0.76	0.69		0.75	0.73	0.66
gb:KF268949		0.76	0.73	0.66		0.70	0.68	0.61		0.73	0.70	0.62		0.78	0.76	0.70		0.75	0.76	0.68		0.75	0.72	0.65
gb:KF268950		0.75	0.73	0.66		0.72	0.69	0.63		0.73	0.70	0.62		0.78	0.76	0.69		0.75	0.76	0.68		0.75	0.73	0.66
**Average**		0.75	0.73	0.66		0.72	0.69	0.62		0.73	0.70	0.62		0.78	0.76	0.69		0.75	0.76	0.68		0.75	0.73	0.66
**Standard deviation**		0.00	0.00	0.00		0.01	0.01	0.01		0.00	0.00	0.01		0.00	0.00	0.00		0.00	0.00	0.01		0.00	0.00	0.00
**ZIKV Coding Sequences**			***C***				***E***				***NS1***				***NS2A***				***NS2B***					
**ZIKV Strains (WA)**		**HS**	**AG**	**AB**		**HS**	**AG**	**AB**		**HS**	**AG**	**AB**		**HS**	**AG**	**AB**		**HS**	**AG**	**AB**				
gb:HQ234501		0.77	0.76	0.71		0.77	0.73	0.67		0.77	0.74	0.68		0.75	0.72	0.65		0.73	0.71	0.65				
gb:HQ234500		0.76	0.76	0.70		0.78	0.75	0.68		0.77	0.73	0.67		0.74	0.72	0.64		0.72	0.68	0.63				
**Average**		0.76	0.76	0.70		0.78	0.74	0.68		0.77	0.73	0.67		0.74	0.72	0.65		0.73	0.70	0.64				
**Standard deviation**		0.01	0.00	0.00		0.00	0.01	0.01		0.00	0.00	0.00		0.00	0.00	0.00		0.01	0.01	0.01				
**ZIKV Coding Sequences**			***NS3***				***NS4A***				***NS4B***				***NS5***				***prM***				**Average (ALL)**	
**ZIKV Strains (WA)**		**HS**	**AG**	**AB**		**HS**	**AG**	**AB**		**HS**	**AG**	**AB**		**HS**	**AG**	**AB**		**HS**	**AG**	**AB**		**HS**	**AG**	**AB**
gb:HQ234501		0.75	0.72	0.65		0.73	0.71	0.64		0.76	0.71	0.63		0.78	0.76	0.69		0.72	0.73	0.67		0.75	0.73	0.67
gb:HQ234500		0.75	0.72	0.65		0.70	0.69	0.62		0.76	0.72	0.64		0.78	0.76	0.70		0.75	0.74	0.67		0.75	0.73	0.66
**Average**		0.75	0.72	0.65		0.71	0.70	0.63		0.76	0.71	0.63		0.78	0.76	0.70		0.73	0.74	0.67		0.75	0.73	0.66
**Standard deviation**		0.00	0.00	0.00		0.01	0.01	0.01		0.00	0.00	0.00		0.00	0.00	0.00		0.01	0.00	0.00		0.00	0.00	0.00

EA: East African. WA: West African. HS: Homo sapiens. AG: Aedes aegypti. AB: Aedes albopictus
