# Supplementary material for: Evolution of codon usage in Zika virus genomes is host and vector specific
Source: Emerg Microbes Infect. 2016 Oct 12;5(10):e107–. doi: 10.1038/emi.2016.106 (PMC5117728; doi:10.1038/emi.2016.106)
Supplement: Supplementary Table S5 [file emi2016106x8.pdf]

Supplementary Table S5. Summary of relative codon deoptimization index (RCDI) analysis.

**ZIKV Coding Sequences**		***C***			***E***			***NS1***			***NS2A***			***NS2B***		
**ZIKV Strains**		**HS**	**AG**	**AB**	**HS**	**AG**	**AB**	**HS**	**AG**	**AB**	**HS**	**AG**	**AB**	**HS**	**AG**	**AB**
KF268948|ARB13565	**EA**	1.35	1.37	1.47	1.21	1.26	1.35	1.20	1.39	1.52	1.21	1.24	1.33	1.33	1.43	1.55
EU545988|FSM	**Asian**	1.22	1.35	1.42	1.19	1.24	1.37	1.21	1.41	1.53	1.18	1.18	1.23	1.49	1.50	1.53
KU681082|CPC-0740	**Asian**	1.22	1.32	1.40	1.18	1.25	1.37	1.22	1.43	1.54	1.18	1.17	1.24	1.46	1.47	1.52
KJ776791|H/PF/2013	**Asian**	1.20	1.27	1.35	1.19	1.24	1.36	1.20	1.39	1.51	1.21	1.16	1.21	1.40	1.44	1.45
KU681081|SV0127/14	**Asian**	1.22	1.27	1.36	1.20	1.24	1.37	1.21	1.39	1.51	1.20	1.19	1.25	1.38	1.44	1.48
KU509998|Haiti/1225/2014	**Asian**	1.22	1.29	1.38	1.20	1.25	1.37	1.20	1.39	1.50	1.19	1.18	1.24	1.40	1.44	1.45
KU365779|BeH819966	**Asian**	1.20	1.27	1.35	1.20	1.25	1.38	1.20	1.39	1.50	1.20	1.17	1.23	1.40	1.44	1.45
KU497555|Brazil-ZKV2015	**Asian**	1.20	1.27	1.34	1.20	1.26	1.39	1.20	1.39	1.51	1.20	1.18	1.23	1.39	1.43	1.44
KU647676|MRS_OPY	**Asian**	1.19	1.26	1.34	1.20	1.25	1.38	1.20	1.40	1.52	1.21	1.19	1.25	1.41	1.45	1.47
KU527068|Natal_RGN	**Asian**	1.22	1.29	1.38	1.20	1.25	1.37	1.19	1.39	1.50	1.20	1.18	1.24	1.40	1.44	1.45
KU707826|SSABR1	**Asian**	1.20	1.27	1.35	1.20	1.25	1.38	1.20	1.39	1.50	1.20	1.17	1.23	1.40	1.44	1.45
KU501215|PRVABC59	**Asian**	1.19	1.27	1.36	1.20	1.25	1.38	1.20	1.39	1.51	1.21	1.18	1.24	1.40	1.44	1.45
KU501216|103344	**Asian**	1.20	1.27	1.35	1.20	1.25	1.38	1.20	1.39	1.51	1.19	1.16	1.22	1.40	1.44	1.45
KU501217|8375	**Asian**	1.20	1.27	1.35	1.20	1.25	1.38	1.20	1.39	1.51	1.19	1.16	1.22	1.40	1.44	1.45
KU365780|BeH815744	**Asian**	1.20	1.27	1.35	1.20	1.25	1.38	1.20	1.39	1.50	1.20	1.17	1.23	1.40	1.44	1.45
KU365778|BeH819015	**Asian**	1.21	1.27	1.36	1.19	1.26	1.38	1.20	1.39	1.50	1.20	1.18	1.24	1.39	1.43	1.45
KU312312|Z1106033	**Asian**	1.20	1.27	1.35	1.19	1.26	1.39	1.19	1.37	1.49	1.20	1.17	1.23	1.40	1.44	1.45
KU365777|BeH818995	**Asian**	1.20	1.27	1.35	1.20	1.25	1.38	1.20	1.39	1.50	1.20	1.17	1.23	1.40	1.44	1.45
KU321639|ZikaSPH2015	**Asian**	1.22	1.30	1.39	1.19	1.25	1.37	1.20	1.39	1.50	1.19	1.18	1.24	1.40	1.44	1.45
AY632535|MR_766	**EA**	1.30	1.34	1.45	1.16	1.21	1.29	1.20	1.37	1.52	1.24	1.24	1.34	1.30	1.38	1.49
KF268949|ARB15076	**EA**	1.38	1.41	1.53	1.21	1.26	1.36	1.20	1.37	1.52	1.18	1.18	1.28	1.33	1.46	1.56
KF268950|ARB7701	**EA**	1.35	1.37	1.47	1.21	1.26	1.35	1.20	1.39	1.52	1.21	1.24	1.33	1.33	1.43	1.55
HQ234501|ArD_41519	**WA**	1.31	1.35	1.41	1.23	1.29	1.39	1.22	1.39	1.48	1.14	1.16	1.24	1.37	1.45	1.55
HQ234500|IbH_30656	**WA**	1.37	1.38	1.44	1.23	1.27	1.38	1.18	1.36	1.46	1.18	1.19	1.28	1.40	1.50	1.61
KF993678|PLCal_ZV	**Asian**	1.40	1.58	1.67	1.19	1.23	1.36	1.21	1.39	1.51	1.20	1.20	1.26	1.44	1.46	1.50
HQ234499|P6-740	**Asian**	1.27	1.27	1.33	1.22	1.23	1.36	1.20	1.38	1.50	1.16	1.16	1.20	1.39	1.49	1.54
JN860885|FSS13025	**Asian**	1.20	1.30	1.37	1.18	1.24	1.36	1.21	1.41	1.53	1.16	1.15	1.21	1.48	1.51	1.55
**Average**		1.25	1.31	1.40	1.20	1.25	1.37	1.20	1.39	1.51	1.19	1.18	1.25	1.39	1.45	1.49
**Standard deviation**		0.07	0.07	0.07	0.01	0.01	0.02	0.01	0.01	0.02	0.02	0.02	0.04	0.04	0.03	0.05

**ZIKV Coding Sequences**		***NS3***			***NS4A***			***NS4B***			***NS5***			***prM***			**Average (ALL)**		
**ZIKV Strains**		**HS**	**AG**	**AB**	**HS**	**AG**	**AB**	**HS**	**AG**	**AB**	**HS**	**AG**	**AB**	**HS**	**AG**	**AB**	**HS**	**AG**	**AB**
KF268948|ARB13565	**EA**	1.13	1.36	1.49	1.36	1.38	1.50	1.23	1.32	1.43	1.04	1.15	1.24	1.30	1.28	1.39	1.24	1.32	1.43
EU545988|FSM	**Asian**	1.12	1.35	1.48	1.41	1.38	1.47	1.17	1.25	1.31	1.04	1.17	1.24	1.24	1.40	1.49	1.23	1.32	1.41
KU681082|CPC-0740	**Asian**	1.13	1.36	1.49	1.43	1.44	1.53	1.17	1.31	1.37	1.03	1.15	1.24	1.24	1.41	1.52	1.23	1.33	1.42
KJ776791|H/PF/2013	**Asian**	1.13	1.36	1.48	1.41	1.44	1.55	1.19	1.29	1.36	1.06	1.17	1.24	1.30	1.42	1.53	1.23	1.32	1.40
KU681081|SV0127/14	**Asian**	1.13	1.36	1.49	1.40	1.39	1.47	1.18	1.27	1.32	1.04	1.16	1.24	1.27	1.40	1.50	1.22	1.31	1.40
KU509998|Haiti/1225/2014	**Asian**	1.13	1.36	1.49	1.41	1.44	1.55	1.17	1.26	1.32	1.06	1.17	1.24	1.31	1.42	1.54	1.23	1.32	1.41
KU365779|BeH819966	**Asian**	1.13	1.37	1.49	1.41	1.44	1.55	1.19	1.29	1.36	1.05	1.17	1.24	1.31	1.42	1.54	1.23	1.32	1.41
KU497555|Brazil-ZKV2015	**Asian**	1.13	1.36	1.49	1.41	1.44	1.55	1.19	1.29	1.36	1.06	1.17	1.24	1.31	1.42	1.54	1.23	1.32	1.41
KU647676|MRS_OPY	**Asian**	1.13	1.36	1.49	1.41	1.44	1.55	1.19	1.30	1.37	1.06	1.17	1.25	1.32	1.43	1.54	1.23	1.32	1.41
KU527068|Natal_RGN	**Asian**	1.13	1.36	1.49	1.41	1.44	1.55	1.20	1.29	1.36	1.06	1.17	1.24	1.31	1.42	1.54	1.23	1.32	1.41
KU707826|SSABR1	**Asian**	1.13	1.36	1.48	1.41	1.44	1.55	1.19	1.29	1.36	1.05	1.17	1.24	1.31	1.42	1.54	1.23	1.32	1.41
KU501215|PRVABC59	**Asian**	1.13	1.37	1.50	1.42	1.44	1.54	1.19	1.30	1.36	1.06	1.17	1.25	1.32	1.42	1.53	1.23	1.32	1.41
KU501216|103344	**Asian**	1.12	1.36	1.49	1.42	1.43	1.54	1.19	1.28	1.35	1.06	1.18	1.25	1.31	1.42	1.54	1.23	1.32	1.41
KU501217|8375	**Asian**	1.13	1.37	1.50	1.42	1.43	1.54	1.20	1.28	1.35	1.06	1.18	1.25	1.31	1.42	1.54	1.23	1.32	1.41
KU365780|BeH815744	**Asian**	1.13	1.36	1.48	1.41	1.44	1.55	1.19	1.29	1.36	1.06	1.17	1.24	1.32	1.44	1.55	1.23	1.32	1.41
KU365778|BeH819015	**Asian**	1.13	1.35	1.48	1.42	1.44	1.54	1.19	1.29	1.35	1.05	1.17	1.24	1.31	1.42	1.54	1.23	1.32	1.41
KU312312|Z1106033	**Asian**	1.12	1.36	1.49	1.41	1.43	1.54	1.19	1.29	1.35	1.06	1.17	1.25	1.34	1.44	1.55	1.23	1.32	1.41
KU365777|BeH818995	**Asian**	1.13	1.36	1.49	1.41	1.44	1.55	1.19	1.29	1.36	1.06	1.17	1.24	1.32	1.44	1.55	1.23	1.32	1.41
KU321639|ZikaSPH2015	**Asian**	1.12	1.34	1.46	1.41	1.44	1.55	1.17	1.26	1.32	1.06	1.17	1.24	1.31	1.42	1.54	1.23	1.32	1.41
AY632535|MR_766	**EA**	1.13	1.34	1.49	1.22	1.35	1.48	1.19	1.26	1.36	1.05	1.18	1.25	1.28	1.30	1.37	1.21	1.30	1.40
KF268949|ARB15076	**EA**	1.12	1.35	1.48	1.38	1.39	1.53	1.25	1.35	1.46	1.05	1.15	1.23	1.24	1.23	1.34	1.23	1.32	1.43
KF268950|ARB7701	**EA**	1.18	1.38	1.53	1.36	1.38	1.50	1.23	1.32	1.43	1.04	1.15	1.24	1.30	1.28	1.39	1.24	1.32	1.43
HQ234501|ArD_41519	**WA**	1.16	1.35	1.48	1.32	1.27	1.39	1.16	1.30	1.40	1.04	1.16	1.23	1.28	1.31	1.36	1.22	1.30	1.39
HQ234500|IbH_30656	**WA**	1.17	1.35	1.50	1.30	1.29	1.39	1.13	1.27	1.37	1.04	1.15	1.22	1.18	1.25	1.31	1.22	1.30	1.40
KF993678|PLCal_ZV	**Asian**	1.16	1.35	1.48	1.41	1.45	1.54	1.21	1.31	1.37	1.04	1.16	1.23	1.29	1.43	1.53	1.25	1.36	1.44
HQ234499|P6-740	**Asian**	1.18	1.36	1.50	1.36	1.38	1.47	1.13	1.22	1.31	1.05	1.18	1.25	1.37	1.51	1.62	1.23	1.32	1.41
JN860885|FSS13025	**Asian**	1.18	1.36	1.50	1.39	1.41	1.49	1.20	1.28	1.35	1.05	1.17	1.24	1.26	1.42	1.52	1.23	1.32	1.41
**Average**		1.14	1.36	1.49	1.39	1.41	1.52	1.19	1.29	1.36	1.05	1.17	1.24	1.30	1.39	1.50	1.23	1.32	1.41
**Standard deviation**		0.02	0.01	0.01	0.05	0.05	0.05	0.03	0.03	0.04	0.01	0.01	0.01	0.04	0.07	0.08	0.01	0.01	0.01

**ZIKV Coding Sequences**	***C***			***E***			***NS1***			***NS2A***			***NS2B***		
**ZIKV Strains (Asian)**	**HS**	**AG**	**AB**	**HS**	**AG**	**AB**	**HS**	**AG**	**AB**	**HS**	**AG**	**AB**	**HS**	**AG**	**AB**
gb:EU545988	1.22	1.35	1.42	1.19	1.24	1.37	1.21	1.41	1.53	1.18	1.18	1.23	1.49	1.50	1.53
gb:KU681082	1.22	1.32	1.40	1.18	1.25	1.37	1.22	1.43	1.54	1.18	1.17	1.24	1.46	1.47	1.52
gb:KJ776791	1.20	1.27	1.35	1.19	1.24	1.36	1.20	1.39	1.51	1.21	1.16	1.21	1.40	1.44	1.45
gb:KU681081	1.22	1.27	1.36	1.20	1.24	1.37	1.21	1.39	1.51	1.20	1.19	1.25	1.38	1.44	1.48
gb:KU509998	1.22	1.29	1.38	1.20	1.25	1.37	1.20	1.39	1.50	1.19	1.18	1.24	1.40	1.44	1.45
gb:KU365779	1.20	1.27	1.35	1.20	1.25	1.38	1.20	1.39	1.50	1.20	1.17	1.23	1.40	1.44	1.45
gb:KU497555	1.20	1.27	1.34	1.20	1.26	1.39	1.20	1.39	1.51	1.20	1.18	1.23	1.39	1.43	1.44
gb:KU647676	1.19	1.26	1.34	1.20	1.25	1.38	1.20	1.40	1.52	1.21	1.19	1.25	1.41	1.45	1.47
gb:KU527068	1.22	1.29	1.38	1.20	1.25	1.37	1.19	1.39	1.50	1.20	1.18	1.24	1.40	1.44	1.45
gb:KU707826	1.20	1.27	1.35	1.20	1.25	1.38	1.20	1.39	1.50	1.20	1.17	1.23	1.40	1.44	1.45
gb:KU501215	1.19	1.27	1.36	1.20	1.25	1.38	1.20	1.39	1.51	1.21	1.18	1.24	1.40	1.44	1.45
gb:KU501216	1.20	1.27	1.35	1.20	1.25	1.38	1.20	1.39	1.51	1.19	1.16	1.22	1.40	1.44	1.45
gb:KU501217	1.20	1.27	1.35	1.20	1.25	1.38	1.20	1.39	1.51	1.19	1.16	1.22	1.40	1.44	1.45
gb:KU365780	1.20	1.27	1.35	1.20	1.25	1.38	1.20	1.39	1.50	1.20	1.17	1.23	1.40	1.44	1.45
gb:KU365778	1.21	1.27	1.36	1.19	1.26	1.38	1.20	1.39	1.50	1.20	1.18	1.24	1.39	1.43	1.45
gb:KU312312	1.20	1.27	1.35	1.19	1.26	1.39	1.19	1.37	1.49	1.20	1.17	1.23	1.40	1.44	1.45
gb:KU365777	1.20	1.27	1.35	1.20	1.25	1.38	1.20	1.39	1.50	1.20	1.17	1.23	1.40	1.44	1.45
gb:KU321639	1.22	1.30	1.39	1.19	1.25	1.37	1.20	1.39	1.50	1.19	1.18	1.24	1.40	1.44	1.45
gb:KF993678	1.40	1.58	1.67	1.19	1.23	1.36	1.21	1.39	1.51	1.20	1.20	1.26	1.44	1.46	1.50
gb:HQ234499	1.27	1.27	1.33	1.22	1.23	1.36	1.20	1.38	1.50	1.16	1.16	1.20	1.39	1.49	1.54
gb:JN860885	1.20	1.30	1.37	1.18	1.24	1.36	1.21	1.41	1.53	1.16	1.15	1.21	1.48	1.51	1.55
**Average**	1.22	1.29	1.38	1.19	1.25	1.37	1.20	1.39	1.51	1.19	1.17	1.23	1.41	1.45	1.47
**Standard deviation**	0.04	0.07	0.07	0.01	0.01	0.01	0.01	0.01	0.01	0.01	0.01	0.01	0.03	0.02	0.03

**ZIKV Coding Sequences**	***NS3***			***NS4A***			***NS4B***			***NS5***			***prM***			**Average (ALL)**		
**ZIKV Strains (Asian)**	**HS**	**AG**	**AB**	**HS**	**AG**	**AB**	**HS**	**AG**	**AB**	**HS**	**AG**	**AB**	**HS**	**AG**	**AB**	**HS**	**AG**	**AB**
gb:EU545988	1.12	1.35	1.48	1.41	1.38	1.47	1.17	1.25	1.31	1.04	1.17	1.24	1.24	1.40	1.49	1.23	1.32	1.41
gb:KU681082	1.13	1.36	1.49	1.43	1.44	1.53	1.17	1.31	1.37	1.03	1.15	1.24	1.24	1.41	1.52	1.23	1.33	1.42

gb:KJ776791	1.13	1.36	1.48	1.41	1.44	1.55	1.19	1.29	1.36	1.06	1.17	1.24	1.30	1.42	1.53	1.23	1.32	1.40
gb:KU681081	1.13	1.36	1.49	1.40	1.39	1.47	1.18	1.27	1.32	1.04	1.16	1.24	1.27	1.40	1.50	1.22	1.31	1.40
gb:KU509998	1.13	1.36	1.49	1.41	1.44	1.55	1.17	1.26	1.32	1.06	1.17	1.24	1.31	1.42	1.54	1.23	1.32	1.41
gb:KU365779	1.13	1.37	1.49	1.41	1.44	1.55	1.19	1.29	1.36	1.05	1.17	1.24	1.31	1.42	1.54	1.23	1.32	1.41
gb:KU497555	1.13	1.36	1.49	1.41	1.44	1.55	1.19	1.29	1.36	1.06	1.17	1.24	1.31	1.42	1.54	1.23	1.32	1.41
gb:KU647676	1.13	1.36	1.49	1.41	1.44	1.55	1.19	1.30	1.37	1.06	1.17	1.25	1.32	1.43	1.54	1.23	1.32	1.41
gb:KU527068	1.13	1.36	1.49	1.41	1.44	1.55	1.20	1.29	1.36	1.06	1.17	1.24	1.31	1.42	1.54	1.23	1.32	1.41
gb:KU707826	1.13	1.36	1.48	1.41	1.44	1.55	1.19	1.29	1.36	1.05	1.17	1.24	1.31	1.42	1.54	1.23	1.32	1.41
gb:KU501215	1.13	1.37	1.50	1.42	1.44	1.54	1.19	1.30	1.36	1.06	1.17	1.25	1.32	1.42	1.53	1.23	1.32	1.41
gb:KU501216	1.12	1.36	1.49	1.42	1.43	1.54	1.19	1.28	1.35	1.06	1.18	1.25	1.31	1.42	1.54	1.23	1.32	1.41
gb:KU501217	1.13	1.37	1.50	1.42	1.43	1.54	1.20	1.28	1.35	1.06	1.18	1.25	1.31	1.42	1.54	1.23	1.32	1.41
gb:KU365780	1.13	1.36	1.48	1.41	1.44	1.55	1.19	1.29	1.36	1.06	1.17	1.24	1.32	1.44	1.55	1.23	1.32	1.41
gb:KU365778	1.13	1.35	1.48	1.42	1.44	1.54	1.19	1.29	1.35	1.05	1.17	1.24	1.31	1.42	1.54	1.23	1.32	1.41
gb:KU312312	1.12	1.36	1.49	1.41	1.43	1.54	1.19	1.29	1.35	1.06	1.17	1.25	1.34	1.44	1.55	1.23	1.32	1.41
gb:KU365777	1.13	1.36	1.49	1.41	1.44	1.55	1.19	1.29	1.36	1.06	1.17	1.24	1.32	1.44	1.55	1.23	1.32	1.41
gb:KU321639	1.12	1.34	1.46	1.41	1.44	1.55	1.17	1.26	1.32	1.06	1.17	1.24	1.31	1.42	1.54	1.23	1.32	1.41
gb:KF993678	1.16	1.35	1.48	1.41	1.45	1.54	1.21	1.31	1.37	1.04	1.16	1.23	1.29	1.43	1.53	1.25	1.36	1.44
gb:HQ234499	1.18	1.36	1.50	1.36	1.38	1.47	1.13	1.22	1.31	1.05	1.18	1.25				1.22	1.30	1.38
gb:JN860885	1.18	1.36	1.50	1.39	1.41	1.49	1.20	1.28	1.35	1.05	1.17	1.24	1.26	1.42	1.52	1.23	1.32	1.41
**Average**	1.13	1.36	1.49	1.41	1.43	1.53	1.19	1.28	1.35	1.05	1.17	1.24	1.30	1.42	1.53	1.23	1.32	1.41
**Standard deviation**	0.02	0.01	0.01	0.01	0.02	0.03	0.02	0.02	0.02	0.01	0.01	0.01	0.03	0.01	0.01	0.01	0.01	0.01
**ZIKV Coding Sequences**		***C***			***E***			***NS1***			***NS2A***			***NS2B***				
**ZIKV Strains (EA)**	**HS**	**AG**	**AB**	**HS**	**AG**	**AB**	**HS**	**AG**	**AB**	**HS**	**AG**	**AB**	**HS**	**AG**	**AB**			
gb:KF268948	1.35	1.37	1.47	1.21	1.26	1.35	1.20	1.39	1.52	1.21	1.24	1.33	1.33	1.43	1.55			
gb:AY632535	1.30	1.34	1.45	1.16	1.21	1.29	1.20	1.37	1.52	1.24	1.24	1.34	1.30	1.38	1.49			
gb:KF268949	1.38	1.41	1.53	1.21	1.26	1.36	1.20	1.37	1.52	1.18	1.18	1.28	1.33	1.46	1.56			
gb:KF268950	1.35	1.37	1.47	1.21	1.26	1.35	1.20	1.39	1.52	1.21	1.24	1.33	1.33	1.43	1.55			
**Average**	1.34	1.37	1.48	1.20	1.25	1.34	1.20	1.38	1.52	1.21	1.23	1.32	1.32	1.42	1.54			
**Standard deviation**	0.03	0.02	0.03	0.02	0.02	0.03	0.00	0.01	0.00	0.02	0.02	0.03	0.01	0.03	0.03			
**ZIKV Coding Sequences**		***NS3***			***NS4A***			***NS4B***			***NS5***			***prM***		**Average (ALL)**		
**ZIKV Strains (EA)**	**HS**	**AG**	**AB**	**HS**	**AG**	**AB**	**HS**	**AG**	**AB**	**HS**	**AG**	**AB**	**HS**	**AG**	**AB**	**HS**	**AG**	**AB**
gb:KF268948	1.13	1.36	1.49	1.36	1.38	1.50	1.23	1.32	1.43	1.04	1.15	1.24	1.30	1.28	1.39	1.24	1.32	1.43
gb:AY632535	1.13	1.34	1.49	1.22	1.35	1.48	1.19	1.26	1.36	1.05	1.18	1.25	1.28	1.30	1.37	1.21	1.30	1.40
gb:KF268949	1.12	1.35	1.48	1.38	1.39	1.53	1.25	1.35	1.46	1.05	1.15	1.23	1.24	1.23	1.34	1.23	1.32	1.43
gb:KF268950	1.18	1.38	1.53	1.36	1.38	1.50	1.23	1.32	1.43	1.04	1.15	1.24	1.30	1.28	1.39	1.24	1.32	1.43
**Average**	1.14	1.36	1.50	1.33	1.37	1.50	1.23	1.31	1.42	1.04	1.16	1.24	1.28	1.27	1.37	1.23	1.31	1.42
**Standard deviation**	0.02	0.01	0.02	0.07	0.02	0.02	0.02	0.03	0.04	0.00	0.01	0.01	0.02	0.02	0.02	0.01	0.01	0.01
**ZIKV Coding Sequences**		***C***			***E***			***NS1***			***NS2A***			***NS2B***				
**ZIKV Strains (WA)**	**HS**	**AG**	**AB**	**HS**	**AG**	**AB**	**HS**	**AG**	**AB**	**HS**	**AG**	**AB**	**HS**	**AG**	**AB**			
gb:HQ234501	1.31	1.35	1.41	1.23	1.29	1.39	1.22	1.39	1.48	1.14	1.16	1.24	1.37	1.45	1.55			
gb:HQ234500	1.37	1.38	1.44	1.23	1.27	1.38	1.18	1.36	1.46	1.18	1.19	1.28	1.40	1.50	1.61			
**Average**	1.34	1.36	1.42	1.23	1.28	1.39	1.20	1.37	1.47	1.16	1.17	1.26	1.38	1.48	1.58			
**Standard deviation**	0.03	0.02	0.02	0.00	0.01	0.01	0.02	0.01	0.01	0.02	0.01	0.02	0.01	0.03	0.03			
**ZIKV Coding Sequences**		***NS3***			***NS4A***			***NS4B***			***NS5***			***prM***		**Average (ALL)**		
**ZIKV Strains (WA)**	**HS**	**AG**	**AB**	**HS**	**AG**	**AB**	**HS**	**AG**	**AB**	**HS**	**AG**	**AB**	**HS**	**AG**	**AB**	**HS**	**AG**	**AB**
gb:HQ234501	1.16	1.35	1.48	1.32	1.27	1.39	1.16	1.30	1.40	1.04	1.16	1.23	1.28	1.31	1.36	1.22	1.30	1.39
gb:HQ234500	1.17	1.35	1.50	1.30	1.29	1.39	1.13	1.27	1.37	1.04	1.15	1.22	1.18	1.25	1.31	1.22	1.30	1.40
**Average**	1.16	1.35	1.49	1.31	1.28	1.39	1.14	1.28	1.38	1.04	1.15	1.23	1.23	1.28	1.33	1.22	1.30	1.39
**Standard deviation**	0.01	0.00	0.01	0.01	0.01	0.00	0.02	0.02	0.02	0.00	0.00	0.01	0.05	0.03	0.02	0.00	0.00	0.00

EA: East African. WA: West African. HS: Homo sapiens. AG: Aedes aegypti. AB: Aedes albopictus
